# A data-driven model of brain volume changes in progressive supranuclear palsy

**DOI:** 10.1093/braincomms/fcac098

**Published:** 2022-04-14

**Authors:** W. J. Scotton, M. Bocchetta, E. Todd, D. M. Cash, N. Oxtoby, L. VandeVrede, H. Heuer, PROSPECT Consortium, 4RTNI Consortium, D. C. Alexander, J. B. Rowe, H. R. Morris, A. Boxer, J. D. Rohrer, P. A. Wijeratne

**Affiliations:** 1 Dementia Research Centre, Department of Neurodegenerative Disease, UCL Queen Square Institute of Neurology, University College London, London, UK; 2 Centre for Medical Image Computing, Department of Computer Science, University College London, London, UK; 3 Department of Neurology, Memory and Aging Center, University of California, San Francisco, CA, USA; 4 Department of Clinical Neurosciences, Cambridge University, Cambridge University Hospitals NHS Trust, Cambridge, UK; 5 Medical Research Council Cognition and Brain Sciences Unit, Cambridge University, Cambridge, UK; 6 Department of Clinical and Movement Neurosciences, University College London Queen Square Institute of Neurology, London, UK; 7 Movement Disorders Centre, University College London Queen Square Institute of Neurology, London, UK

**Keywords:** event-based model, disease progression, progressive supranuclear palsy, biomarkers, machine learning

## Abstract

The most common clinical phenotype of progressive supranuclear palsy is Richardson syndrome, characterized by levodopa unresponsive symmetric parkinsonism, with a vertical supranuclear gaze palsy, early falls and cognitive impairment. There is currently no detailed understanding of the full sequence of disease pathophysiology in progressive supranuclear palsy. Determining the sequence of brain atrophy in progressive supranuclear palsy could provide important insights into the mechanisms of disease progression, as well as guide patient stratification and monitoring for clinical trials. We used a probabilistic event-based model applied to cross-sectional structural MRI scans in a large international cohort, to determine the sequence of brain atrophy in clinically diagnosed progressive supranuclear palsy Richardson syndrome. A total of 341 people with Richardson syndrome (of whom 255 had 12-month follow-up imaging) and 260 controls were included in the study. We used a combination of 12-month follow-up MRI scans, and a validated clinical rating score (progressive supranuclear palsy rating scale) to demonstrate the longitudinal consistency and utility of the event-based model’s staging system. The event-based model estimated that the earliest atrophy occurs in the brainstem and subcortical regions followed by progression caudally into the superior cerebellar peduncle and deep cerebellar nuclei, and rostrally to the cortex. The sequence of cortical atrophy progresses in an anterior to posterior direction, beginning in the insula and then the frontal lobe before spreading to the temporal, parietal and finally the occipital lobe. This *in vivo* ordering accords with the post-mortem neuropathological staging of progressive supranuclear palsy and was robust under cross-validation. Using longitudinal information from 12-month follow-up scans, we demonstrate that subjects consistently move to later stages over this time interval, supporting the validity of the model. In addition, both clinical severity (progressive supranuclear palsy rating scale) and disease duration were significantly correlated with the predicted subject event-based model stage (*P* < 0.01). Our results provide new insights into the sequence of atrophy progression in progressive supranuclear palsy and offer potential utility to stratify people with this disease on entry into clinical trials based on disease stage, as well as track disease progression.

## Teaser

Using event-based modelling, *Scotton et al.* report the probabilistic sequence of brain atrophy progression in progressive supranuclear palsy. The resulting data-driven model, which is correlated with both clinical severity and disease duration, allows individual patient staging at baseline MRI scan with potential utility for clinical trial enrichment.

## Graphical Abstract

**Graphical Abstract:**
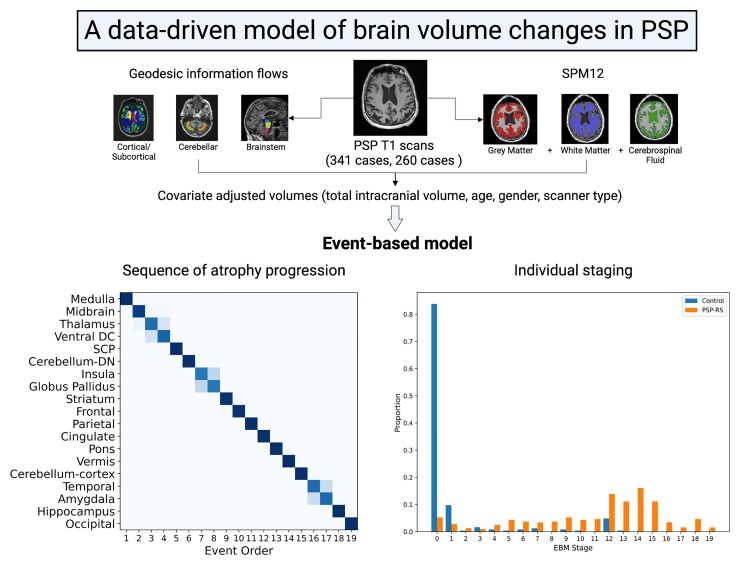

See Günter Höglinger (https://doi.org/10.1093/braincomms/fcac113) for a scientific commentary on this article.

## Introduction

Progressive supranuclear palsy (PSP) is a severe neurodegenerative condition, with an estimated prevalence of 5–7 per 100 000 and survival of just 5–7 years.^1,2^ PSP pathology can present with a range of clinical phenotypes involving language, behavioural and movement abnormalities.^3^ This heterogeneity in clinical presentation has been operationalized in the Movement Disorder Society 2017 PSP diagnostic criteria.^4^ The most common clinical phenotype is PSP Richardson syndrome (PSP-RS), similar to the cases first described by Steele *et al.*,^5^ and characterized by a levodopa unresponsive parkinsonian syndrome with a vertical supranuclear gaze palsy, early falls and dementia. Natural history studies of PSP-RS have shown the mean age of symptom onset is between 65 and 67 years with an average survival from disease onset of 6–7 years.^2,6^ PSP pathology is characterized by insoluble aggregates of the 4-repeat (4R) isoform of the microtubule-associated protein tau in neurons and glia, predominantly in the subthalamic nucleus (STN), globus pallidus (GP), striatum, the dentate nucleus of the cerebellum, frontal lobes and to a lesser extent in the occipital cortices.^7^ The recent pathology staging system for PSP defines six sequential stages of progression, starting with the STN, spreading out caudally to the cortex and rostrally to the cerebellum.^8^ This has been validated in an independent cohort with increasing pathological stage correlating with clinical severity.^9^

No effective disease-modifying treatment has yet been proven for PSP, despite recent successful clinical trials.^10,11^ Clinical trials in PSP can be complicated by variable disease stage at trial entry, highlighting the importance of stratifying patients into homogenous cohorts based on disease stage with similar rates of disease progression. Although the PSP rating scale has been shown to be a good independent predictor of survival,^12^ and is used as the primary end-point in clinical trials, such clinical biomarkers are only indirect measures of the biological stage of disease and are affected by intra- and inter-rater variability, as well as fluctuation in patients’ clinical state. Reliable and individualized disease progression markers are, therefore, required to complement clinical ratings scales.^13^

Structural MRI reveals significant atrophy in the brainstem and subcortical structures in PSP-RS, with additional involvement of the cortical structures.^14^ Increased rates of atrophy in these regions can be detected over a 12-month period,^15,16^ offering a potential biomarker readout for clinical trials. Although there are new tau-PET tracers emerging that show potential in the 4R tauopathies, these are not yet validated for use in the clinic setting,^17,18^ and in the absence of a validated tau-PET tracer for PSP, structural MRI offers an indirect measure of underlying tau pathology *in vivo*. Indeed, a previous study in PSP showed that *in vivo* structural imaging reflected the independent contributions from tau burden and neurodegeneration at autopsy,^19^ while the link in Alzheimer’s disease is well established.^20,21^ However, the order in which brain regions show evidence of increased atrophy *in vivo* is currently unknown.

One approach to estimating the sequence of atrophy progression is event-based modelling (EBM),^22^ using a probabilistic data-driven generative model to infer the order in which biomarkers become abnormal. The EBM can be built from cross-sectional data by combining severity information across biomarkers and individuals without reference to a given individual’s clinical status.^23^ The EBM allows inference of longitudinal information about disease progression by assuming there is a monotonic progression of an individual biomarker from normal to abnormal (even if this progression is non-linear), so that in a patient cohort containing a spectrum of disease stages, more individuals will necessarily show abnormality in a biomarker that changes early in the disease course. This approach has been successfully applied to Huntington’s disease,^23^ sporadic and familial Alzheimer’s disease,^24–26^ Parkinson’s disease,^27^ multiple sclerosis,^28^ the posterior cortical atrophy variant of Alzheimer’s disease^29^ and amyotrophic lateral sclerosis,^30^ providing a simple and validated method to investigate temporal disease patterns and estimate individuals’ disease stage. Recent work has demonstrated the clinical utility of the EBM for screening patients on entry into clinical trials, to improve cohort homogeneity and increase the power to detect a treatment effect.^31^

The aim of this study was to define the progression of brain atrophy in clinically diagnosed PSP-RS by developing an EBM that takes cross-sectional structural MR imaging as input. We hypothesized that there is a consistent sequence in which brain regions become atrophic in PSP-RS, in keeping with the recent PSP pathology staging system proposed by Kovacs *et al.*,^8^ and predicted that the image-based EBM stage would be correlated with clinical disease severity as measured by the PSP rating scale.

## Materials and methods

### Subjects

Data from individuals with a clinical diagnosis of possible or probable PSP-RS were collected from six main sources for inclusion in the study: the 4R Tauopathy Imaging Initiative (4RTNI; ClinicalTrials.gov: NCT01804452),^16,32^ the davunetide randomized control trial (DAV; ClinicalTrials.gov: NCT01056965),^33^ the salsalate clinical trial (SAL; ClinicalTrials.gov: NCT02422485),^34^ the young plasma clinical trial (YP; ClinicalTrials.gov: NCT02460731),^34^ the PROgressive Supranuclear Palsy CorTico-Basal Syndrome Multiple System Atrophy Longitudinal Study (PROSPECT; ClinicalTrials.gov: NCT02778607) and the University College London Dementia Research Centre (UCL DRC) FTD cohort. Control data were collected from three sources: the Frontotemporal Lobar Degeneration Neuroimaging Initiative dataset (FTLDNI; http://4rtni-ftldni.ini.usc.edu/) PROSPECT and the UCL DRC FTD cohort. Controls were defined as no known diagnosis of a neurological or neurodegenerative condition and no known history of memory complaints. Further details on individual cohorts are included in the Supplementary material, and a summary of the demographics of each cohort is included in Supplementary Table 1. Appropriate ethics was applied for and approved via the relevant trial and research ethics committees. For inclusion in this study, all patients had to have, as a minimum, a baseline T_1_-weighted volumetric MRI on a 1.5 or 3 T scanner, with basic demographic data (age at time of scan, gender), and disease duration at time of the scan (time from symptom onset to MRI scan). Twelve-month follow-up scans, if available, were also included in the study, as were PSP rating scale scores. Given original trial analyses failed to show any treatment effect (including no change in volumetric MRI measurements) in the davunetide,^33^ salsalate and young plasma trials,^34^ we combined data from each study’s treatment and placebo groups. Longitudinal data (both 12-month follow-up MRI and PSP rating scale) were used for validation of the staging system produced by the baseline EBM.

### Magnetic resonance imaging

Raw volumetric T_1_-weighted MRI images were all processed by the same pipeline. Scans first underwent visual quality control (QC) to ensure correct acquisition and the absence of major artefacts. Next, raw images that passed QC were bias field corrected for magnetic field inhomogeneity, and the whole brain (cortical and subcortical structures) parcellated using the geodesic information flow (GIF) algorithm.^35^ This automatically extracts regions based on the Neuromorphometrics atlas (Neuromorphometrics, Inc.), using an atlas propagation and label fusion strategy.^36,37^ Subregions of the cerebellum were then automatically extracted with GIF based on the Diedrichsen cerebellar atlas: the cerebellar lobules (I–IV, V, VI, VIIa-Crus I, VIIa-Crus II, VIIb, VIIIa, VIIIb, IX and X), the vermis and the deep nuclei (dentate, interposed and fastigial).^35,38^ The whole brainstem, medulla, pons, superior cerebellar peduncles (SCPs) and midbrain were subsequently segmented using a customized version of the module available in FreeSurfer to accept the GIF parcellation as input for Freesurfer.^39^ Total intracranial volume (TIV) was calculated using SPM12 v6225 (Statistical Parametric Mapping, Wellcome Trust Centre for Neuroimaging, London, UK) running under MATLAB R2012b (Math Works, Natick, MA, USA).^40^ All segmentations were visually inspected to ensure accurate segmentation.

### Biomarker selection

In this study, we use the term biomarker to refer to image-based regional brain volumes that show a significant difference between cases and healthy controls (HCs) (two-tailed *t*-test of mean difference in covariate-adjusted volumes). Given the focus of this study was to test the hypothesis that the sequence of atrophy in PSP-RS is in keeping with the sequence of tau pathology at post-mortem as shown by Kovacs *et al.*,^8^ 19 regions of interest (ROIs) were chosen for inclusion that most closely matched those used in their study; four brainstem (medulla, pons, SCP and midbrain), three cerebellar (cerebellar cortex, deep nuclei and vermis), seven subcortical [thalamus, GP, striatum (caudate and putamen), ventral diencephalon (DC), thalamus, hippocampus and amygdala] and five cortical (frontal, insula, temporal, parietal and occipital) regions. Regions that had a right and left label were combined. All ROIs were controlled for the following covariates using linear regression on the control cohort: age at scan, sex, scanner type and TIV. Linear regressions of age against predicted EBM stage were also performed (after EBM model fitting) for cases and controls separately to confirm that there was no residual correlation after adjustment. All regions selected for inclusion showed a significant difference in covariate-adjusted volumes between cases and controls (Bonferroni-corrected threshold of *p* < 3 × 10^−3^) under a two-tailed *t*-test.

### The event-based model

The EBM is designed to infer a data-driven, probabilistic sequence in which biomarkers become abnormal from cross-sectional data. The strengths of the EBM are firstly that it requires no a priori biomarker cut-offs (thresholds) to define abnormality; secondly, it requires no a priori staging and finally it can produce meaningful results using only moderately sized cross-sectional data. Its reliability with moderately sized data sets makes it ideally suited for analysing biomarkers in rare diseases such as the primary tauopathies.

The EBM is based on the assumptions of homogeneous disease progression and monotonicity: that is all patients have a broadly similar disease progression pattern with a unimodal distribution of orderings, and biomarker change is unidirectional from normal to abnormal i.e. no remission. An ‘event’ is considered to have occurred when a biomarker (in this study an MRI-derived regional volume), has an abnormal value (‘atrophy’) in comparison with the expected values measured in HCs. The model then estimates the sequence *S* = *S*(1), *S*(2), …, *S*(*l*) in which the biomarkers become abnormal, where *S*(1) is the first biomarker and *S*(*l*) is the last. Conceptually, if biomarker A is usually abnormal when biomarker B is abnormal, but B is often abnormal without A, we infer that B occurs before A in the sequence.

The estimation procedure first fits a mixture model to control and patient data for each biomarker. In this study, we decided to use a recent version of the EBM that incorporates a non-parametric method, kernel density estimation (KDE),^29^ for estimating the mixture models. This approach has been shown to perform at a similar level to the classic EBM (that incorporates Gaussian mixture modelling) with parametric input data, while demonstrating superiority when the data are skewed.^29^ The mixture model obtains models for the distribution of normal and abnormal values for each biomarker, providing likelihoods *P*(*x*_*ij*_|*E*_*i*_) and *P*(*x*_*ij*_|¬*E*_*i*_) of observing the value, *x*_*ij*_, of biomarker *i* for subject *j*, given that biomarker *i* has or has not become abnormal, respectively. The EBM combines these likelihoods to then calculate the likelihood of the full data set *X* = *x*_*ij*_: *i* = 1, …, *Z*; *j* = 1, …, *N* for a given sequence, *S*:

(1) $$P(X|S)=\overset{N}{\underset{\;j=1}{\prod }}\;\left[\overset{Z}{\underset{k=0}{\sum }}\;\left(P(k)\overset{k}{\underset{i=1}{\prod }}\;P(x_{ij}|E_{i})\overset{Z}{\underset{i=k+1}{\prod }}\;P(x_{ij}|E_{i})\right)\right]$$

*j* iterates over the number of subjects *N* and *i* iterates over the number of events *Z*. *P*(*k*) refers to the prior likelihood of being at stage *k* and in the absence of prior information is treated as uniform to impose as little information as possible on estimated orderings. The estimation procedure then searches for the characteristic ordering, $\overset{\prime }{S}$, which is the sequence that maximizes the likelihood of *P*(*X*|*S*) in equation (1).^23^ This is found through a combination of a multiply initialized greedy ascent and Markov Chain Monte Carlo (MCMC) sampling, which samples from the posterior distribution on *S*, to find $\overset{\prime }{S}$, which is simply the sequence with the highest (maximum) likelihood. The set of samples from the MCMC sampling also provides information on the uncertainty of the maximum likelihood sequence, which can be visualized on a positional variance diagram.^22,23^

### Patient staging

Once the characteristic sequence, *S*, has been obtained via the EBM, an individual sample *X*_*j*_ (a vector of all measurements across biomarkers *i* for a patient *j*), can be staged by evaluating the stage *k* that maximizes the likelihood in equation (2) below^25^:

(2) $$\begin{array}{rl} {\mathrm{argmax}}_{k}P\left(X_{j}|\overset{\prime }{S},k\right) & ={\mathrm{argmax}}_{k}P(k)\overset{k}{\underset{i=1}{\prod }}\;P(x_{ij}|E_{i})\\ & \;\times \overset{Z}{\underset{i=k+1}{\prod }}\;P(x_{ij}|\neg E_{i}) \end{array}$$

As before *P*(*k*), the prior likelihood of being at stage *k*, is treated as uniform i.e. no a priori information on a particular stage. The EBM stage (*Z*), between 1 and the number of biomarkers, *l*, of subject *j*, is, therefore, given by the stage *k* that maximizes equation (2). Each subject (case and control) had their EBM-predicted stage calculated for their baseline MRI scan and for those that had them, their 12-month follow-up scan.

### Cross-validation of event sequence

Although the MCMC sampling gives some information on the uncertainty of the event ordering in ordering of events derived from the EBM, previous work shows it tends to underestimate this uncertainty.^25^ Bootstrapping is an additional method that tends to give a more liberal estimate of the uncertainty in the ordering. We first performed cross-validation of the maximum likelihood sequence generated by the EBM, by re-estimating the model on 100 bootstrap samples of the original data (sampling with replacement). We then performed repeated stratified 5-fold cross-validation as an additional check on the robustness of the model. This involved refitting the model on 80% of the cohort data and testing accuracy on the held out 20% for each of 10 5-fold random partitions, giving a total of 50 cross-validation folds/models, which are averaged to find the final model sequence.

### Longitudinal validation

We investigated the longitudinal consistency of the staging produced by the EBM, based on the predictions that, firstly, given PSP is a progressive disease, the EBM stage should increase over time, and secondly that increasing EBM stage should be associated with both increasing PSP rating scale score (the main clinical measure of disease severity) and also disease duration, especially during later model stages where there is more widespread atrophy. We staged patients using the baseline EBM based on their 12-month follow-up scan (255 cases) and compared this with predicted stage based on their baseline scan. The follow-up data were processed using the same pipeline as the baseline scans to produce the same ROI biomarkers at 12 months. To test for the relationship of PSP rating scale score with baseline EBM stage, a linear mixed effects model was fit to the data using the lme4 package^41^ in R Studio (version 1.4.1106), with EBM-defined stage as the independent variable and PSP rating scale score as the dependent variable. Two hundred and forty-one baseline and 232 12-month follow-up scans (473 total) had a corresponding PSP rating scale score. Subject Id was modelled as a random effect (random intercept) due to some subjects having two MRI scans at different time points. Significance was calculated using the lmerTest package^42^ which applies Satterthwaite’s method to estimate degrees of freedom and generate *P*-values for mixed models. In addition, we analysed disease duration (time from first symptom to MRI scan) as a function of predicted EBM stage (87 baseline and 43 12-month follow-up scans had disease duration recorded) using the same method. To confirm that baseline EBM stage was also correlated with both PSP-RS score and disease duration we fitted a linear model for each as a function of EBM stage.

### Data availability

Source data are not publicly available but non-commercial academic researcher requests may be made to the Chief Investigators of the six source studies, subject to data access agreements and conditions that preserve participant anonymity. The underlying EBM code is publicly available at https://github.com/noxtoby/kde_ebm.

## Results

### Subject characteristics

Table 1 summarizes the key demographic data for the cohort included in the study. 929 MRI images were processed from a total of 654 subjects: 365 with a clinical diagnosis of PSP-RS (of which 275 had 12-month follow-up scans) and 289 controls. Of the PSP-RS cases, 26 (8%) had a pathological diagnosis after coming to post-mortem: 24 (92%) showed tau pathology consistent with PSP, whereas 2 cases had non-PSP tau pathology [one corticobasal degeneration (CBD) and one globular glial tauopathy (GGT)] and were, therefore, excluded from the analysis. After stringent QC with visual inspection of all images for the remaining 363 cases (pre- and post-processing), 341 PSP-RS cases (of which 255 had 12-month follow-up scans) and 260 control scans were included for the analysis. Reasons for scans failing QC included poor quality of the raw T_1_ image (usually due to movement artefacts) or inaccurate segmentations with the GIF or/and SPM algorithms. Around 70% (241/341) of the cases included had a PSP rating scale score at baseline and follow-up, as well as recorded age, gender, scanner type and TIV. At baseline, the PSP-RS cohort had an older average age [67.9 years, standard deviation (SD) ± 6.8] compared with HCs (62.8 years, SD ± 9.4, *t* = −7.4, *p* < 0.01). Disease duration data [time from diagnosis to baseline visit (average years,  ± SD)] was available for 87 of 341 cases and showed an average length of 4.1 years (SD ± 3.1). There was a higher proportion of females in the control group compared with the PSP-RS group (male/female, 112/148 versus 176/165, respectively, *χ*^2^ = 4.3, *p* = 0.04).

**Table 1:** PSP-RS EBM baseline demographics

Baseline demographics	PSP-RS	Controls	*P-*value
*N* (12 months)	365 (275)	289	–
Post-QC—*N* (12 months)	341 (255)	260	–
Gender (M/F)	176/165	112/148	0.03^a^
Age at first MRI [years (SD)]	67.9 (6.8)	62.8 (9.4)	<0.001^b^
Time symptom onset to first MRI [years (SD)]	4.1 (3.1)	–	–
Pathology (% PSP)	24 (92%)^c^	–	–
PSP rating scale (SD)	38.9 (12.9)^d^	–	–
UPDRS (SD)	30.6 (15.1)	–	–
MOCA (SD)	20.7 (5.1)	–	–

PSP-RS, progressive supranuclear palsy Richardson syndrome.

^a^ *χ* ^2^.

^b^ Unpaired two-tailed *t*-test.

^c^ % of all cases pre-QC.

^d^ 70% (241/341) of baseline cases included had a PSP rating scale score.

### Sequence of atrophy progression

Supplementary Figure 1 shows histograms of the HC and covariate-adjusted PSP-RS ROI biomarker distributions, with KDE mixture model fits and line showing probability of an event. These fits provide the parameters for the normal and abnormal likelihoods, *P*(*x*_*ij*_|*E*_*i*_) and *P*(*x*_*ij*_|¬*E*_*i*_), respectively, that are then used to calculate the maximum likelihood sequence of the full data set. At baseline, all 19 ROI selected for inclusion in the model showed a significantly smaller covariate-adjusted volume in PSP-RS compared with controls.

**Figure 1:**
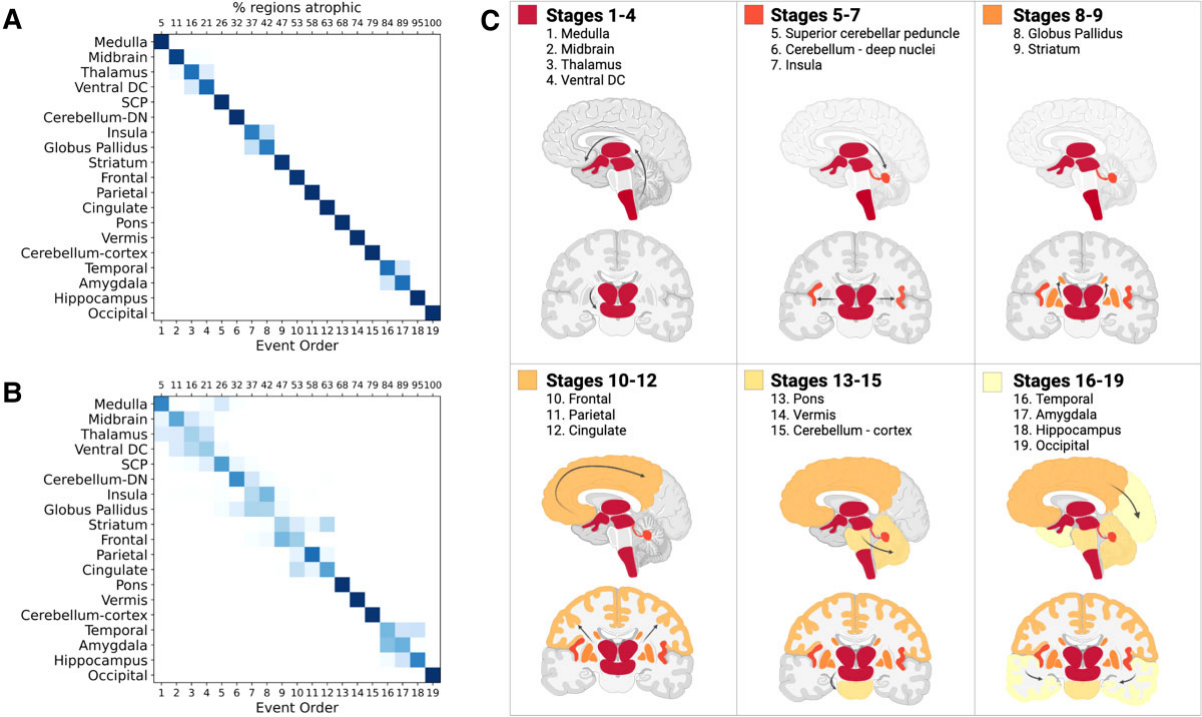
**Sequence of atrophy progression in PSP-RS**. (**A**) Regional volume biomarker positional variance diagram showing the sequence of atrophy progression in PSP-RS. (**B**) Re-estimation of positional variance after cross-validation of the maximum likelihood event sequence by bootstrap resampling (100 bootstraps). For (**A**) and (**B**), the vertical ordering on the *y*-axis (from top to bottom) shows the maximum likelihood sequence estimated by the EBM (earliest to latest event). The bottom *x*-axis shows EBM stage while the top *x*-axis represents the percentage of regions atrophic (abnormal) at each stage. Colour intensity of the squares represents the posterior confidence in each biomarker’s position in the sequence, from either (**A**) MCMC samples of the posterior or (**B**) bootstrapping. SCP, superior cerebellar peduncle; ventral DC, ventral diencephalon. Note that because these volumes are covariate-adjusted the control distribution will be centred at zero. (**C**) Graphic representation of the event sequence with relevant region transitioning from healthy (grey) to unhealthy (coloured). Dark red denotes first regions to atrophy, light yellow denotes last regions to atrophy. Created with BioRender.com.

The positional variance diagram in Fig. 1A shows the most likely sequence in which these regions become atrophic, as estimated by the EBM, as well as the uncertainty in this sequence (based on MCMC sampling of the posterior distributions). The maximum likelihood sequence was estimated using PSP-RS cases only, based on the rationale that PSP is a rare disease, and it is very unlikely for our cohort of controls to have asymptomatic PSP. Indeed, it is more likely the controls would have a common disorder such as Alzheimer’s disease rather than PSP, and we did not want this to confound the sequence estimation hence the exclusion. The EBM estimated that the earliest atrophy occurs in the brainstem and subcortical regions followed by progression caudally into the SCP and deep cerebellar nuclei and rostrally to the cortex. The sequence of cortical atrophy progresses in an anterior to posterior direction, beginning in the insula and then frontal lobe before spreading to the temporal, parietal and finally the occipital lobe (Fig. 1C). The high colour intensity of each square and their presence predominantly on the diagonal of the positional variance diagram indicates that the model has a high degree of certainty regarding their positions in the overall sequence.

### Cross-validation of event sequence

Figure 1B shows positional variance of the maximum likelihood sequence re-estimated by bootstrapping of the data (random resampling with replacement 100 times) and refitting the model. The positional variance diagram for the bootstrapped results represents the proportion of bootstrap samples in which the event *i* (*y*-axis) appears at position *k* (*x*-axis) of the maximum likelihood sequence. The sequence ordering is generally preserved, although as one would expect with this more conservative estimate of uncertainty, there is increased uncertainty in the relative positions early in the sequence from Stage 2 (midbrain) to Stage 4 (ventral DC), and in the middle from Stage 9 (striatum) to Stage 13 (pons). Using repeated stratified 5-fold cross-validation (Supplementary Fig. 2) as an alternative method to assess model robustness (both in terms of the sequence and uncertainty in the sequence), the maximum likelihood sequence is preserved with similar uncertainty in relative positions when visually compared with the bootstrapping method (Fig. 1B).

### Patient staging

**Figure 2:**
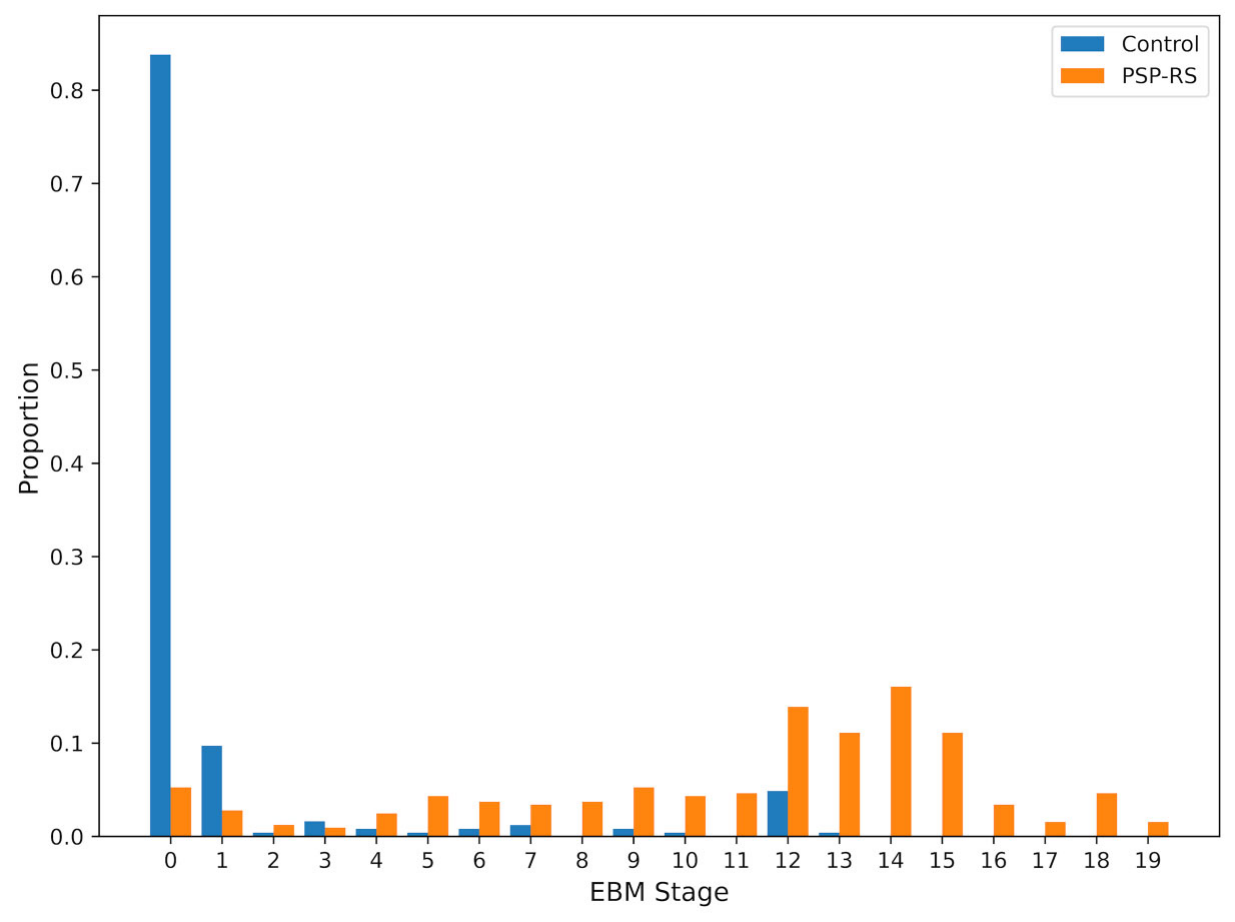
**Histogram of event-based model staging results for PSP-RS.** Healthy controls in blue and PSP-RS cases in orange. Each bar represents the proportion of patients in each category at each EBM stage. Each EBM stage on *x*-axis represents the occurrence of a new biomarker transition event. Stage 0 corresponds to no events having occurred and Stage 19 corresponds to all events having occurred. Events are ordered by the maximum likelihood sequence for the whole PSP-RS population as shown in Fig. 1A.

Figure 2 shows the proportion of subjects at each EBM-defined stage (PSP-RS and HC). Patient staging results were evaluated using the maximum likelihood sequence (Fig. 1A) of regional atrophy for PSP-RS subjects as described in the Methods section. As one would expect the HC cohort is clustered at the early stages with >80% at Stage 0 (i.e. no event occurred), while the PSP-RS cases are distributed more evenly across stages with the highest proportion in the middle to late stages. This suggests that the cohort of PSP cases gathered from multiple different studies were temporally heterogeneous which supports the importance of accurately staging patients using objective biomarkers.

Using a threshold of Stage 2 (medulla and midbrain atrophic) the model was able to correctly classify subjects as PSP-RS versus HC with an overall accuracy of 90% (with a sensitivity and specificity of 91% and 90%, respectively). Although not the focus of this model, the high classification accuracy provided by the EBM further demonstrates its clinical validity.

Outliers were present in both the HC and PSP-RS groups: specifically, 10 (4%) of PSP-RS cases were at Stage 0, whereas 14 controls were at Stage 10 or greater (5%). Visual inspection of these HCs suggested that the segmentations were accurate, but that there were non-specific covariate-adjusted decreased volumes in regions including the hippocampus with relative sparing of the brainstem and subcortical structures, suggesting that these could potentially represent people with preclinical Alzheimer’s disease.

### Longitudinal consistency

**Figure 3:**
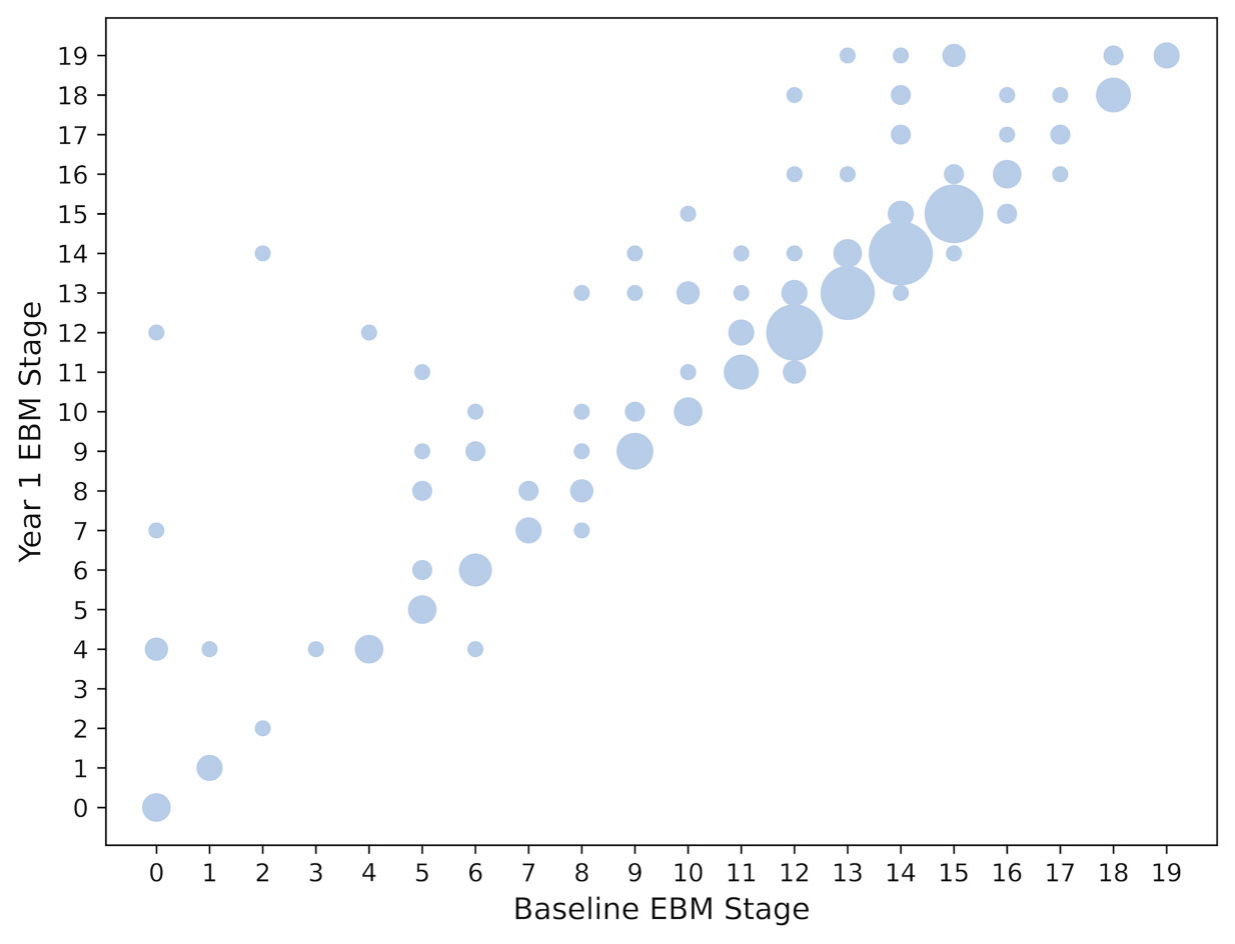
**Longitudinal consistency of baseline EBM.** Scatter plot showing predicted stage at baseline (*x*-axis) versus predicted stage at 12 months (*y*-axis) for those PSP-RS subjects with a follow-up scan (*n* = 255). The area of a circle is weighted by the number of subjects at each point.

To test the validity of the EBM, we first tested the hypothesis that a valid model will produce non-decreasing disease stages for individuals from baseline to follow-up, within the bounds of model uncertainty. Figure 3 compares each PSP-RS subject’s EBM stage at baseline with their stage at 12-month follow-up (255 cases had both a baseline and 12-month follow-up scan). Overall, on this metric the EBM shows good longitudinal consistency with each subjects EBM stage generally increasing or remaining stable at 12-months follow-up: 245/255 cases (96%) either stayed at the same stage or progressed. For these cases, the average stage progression over 12 months was 1 stage. Of the 10 PSP cases that reverted in stage, nine only dropped one stage whereas one dropped two stages.

**Figure 4:**
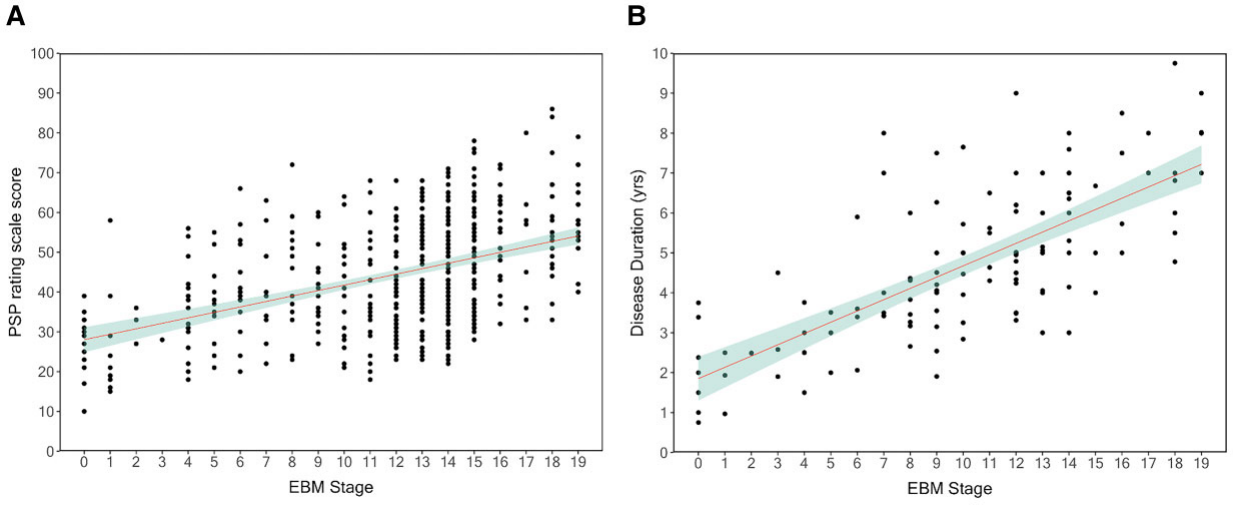
**Association between predicted EBM stage, PSP rating scale score and disease duration**. (**A**) PSP rating scale score versus EBM stage* (*β* = 1.46, 95% CI: 1.2–1.8, *P* < 0.001, conditional *R*^2^ of 0.56 (marginal 0.22) (**B**) Disease duration (years) versus EBM stage** (*β* = 0.29, 95% CI: 0.24–0.34, *P* < 0.001 and a conditional *R*^2^ of 0.68 (marginal 0.41). For both (**A**) and (**B**), the line represents the linear fixed effect model fit to all subjects, and 95% CIs. Subject Id was modelled as a random effect (random intercept) due to some subjects having two MRI scans at different time points. Significance was calculated using Satterthwaite’s method to estimate degrees of freedom and generate *P*-values. * 473 scans (241 baseline and 232 12-month follow-up) with PSP-RS score. ** 130 scans (87 baseline and 43 12-month follow-up) with disease duration.

To further validate the EBM, we first modelled PSP rating scale as a function of predicted EBM stage using a linear mixed model (Fig. 4A). EBM stage was modelled as a fixed effect, whereas Subject Id was modelled as random effect due to some subjects having two MRI scans at different time points. We found a significant fixed effect of EBM stage on predicted PSP rating scale (*β* = 1.46, 95% CI: 1.2–1.8, *P* < 0.001) and a conditional *R*^2^ of 0.56. We then modelled disease duration (years) as a function of predicted EBM stage, which showed a significant fixed effect (*β* = 0.29, 95% CI: 0.24–0.34, *P* < 0.001) and a conditional *R*^2^ of 0.68 (Fig. 4B). When fitting linear models for PSP-RS score and disease duration versus predicted EBM stage on baseline scans only (Supplementary Fig. 3A and B, respectively), there was also a significant association albeit with a lower adjusted *R*^2^ (PSP-RS versus EBM stage at baseline: *β* = 1.14, 95% CI: 0.84–1.44, *P* < 0.001), adjusted *R*^2^ of 0.18, disease duration versus EBM stage at baseline: (*β* = 0.25, 95% CI: 0.20–0.30, *P* < 0.001, adjusted *R*^2^ of 0.39). To check that we had adequately adjusted for age we also ran linear models of age as a function of predicted EBM stage for cases (Supplementary Fig. 4A) and controls separately (Supplementary Fig. 4B). There was no association between EBM stage and age in either the case (*β* = 0.19, 95% CI: 0.13–0.25, *P* = 0.12, adjusted *R*^2^ of 0.017) or control group (*β* = −0.27, 95% CI: −0.66–0.12, *P* = 0.18, adjusted *R*^2^ of 0.003).

## Discussion

The principal result of this study is that a probabilistic data-driven method reveals, *in vivo*, the sequence in which brain regions become atrophic in PSP-RS. We established this sequence from cross-sectional data and went on to demonstrate the validity of this model longitudinally. Ninety-six per cent remained in the same stage or progressed to a later stage over 12 months. The model derived staging correlated with both clinical severity and disease duration.

### Ordering of biomarkers

The order of regional atrophy revealed by the EBM (Fig. 1) broadly mirrors the sequential spread of tau pathology in PSP proposed by Kovacs *et al.*^8^ The earliest atrophy in our model occurs in the brainstem and subcortical regions followed by progression caudally into the SCP and deep cerebellar nuclei and rostrally to the cortex. The sequence of cortical atrophy progresses in an anterior to posterior direction, beginning in the frontal lobe before then spreading to the temporal, parietal and finally the occipital lobe. In the absence of external data to validate the model, we explored the generalizability and robustness of the model using two different validation methods: bootstrap cross-validation and 5-fold repeated stratified 5-fold cross-validation. These demonstrate that even with a more conservative estimate of uncertainty, the sequence of atrophy is largely conserved (Fig. 1B and Supplementary Fig. 2). There remains uncertainty early on between the relative positions of the midbrain, thalamus, ventral DC and SCP, in the middle between the striatum, frontal, parietal, and cingulate lobes, and the pons, and at the end of the sequence between the temporal lobe, amygdala and hippocampus. This heterogeneity is of interest and a motivation for future work.

It is difficult, however, to make a direct comparison between our *in vivo* findings and post-mortem tau histopathology staging for two reasons: first, in this study, we are measuring atrophy rather than tau pathology directly, and although there is evidence that atrophy on structural imaging is associated with tau pathology,^19,20^ it is unlikely to directly correlate with histopathological scores of tau accumulation across neuronal and glial cell populations. Second, two of the regions identified to have the earliest tau pathology in Kovacs’ study are the STN and the substantia nigra (SN), regions that are not individually segmented by the GIF algorithm used in this study. These are subsumed within the ventral DC segmentation in the neuromorphometrics atlas, along with the hypothalamus. Although not specific for the STN and SN, reassuringly this region does occur early in the sequence (Fig. 1A), and after cross-validation one can see (Fig. 1B and Supplementary Fig. 2) that after the medulla there is uncertainty as to the exact ordering of the midbrain, thalamus and ventral DC.

The majority of cross-sectional imaging studies in PSP-RS have focused on the clinical utility of structural MRI as a diagnostic biomarker to differentiate PSP from both Parkinson's disease and other atypical parkinsonian disorders.^13^ These studies usually only give a group-level overview of regional atrophy at baseline, as opposed to the sequence of atrophy changes that we have demonstrated in this study. Even so midbrain atrophy is commonly seen in PSP-RS at baseline, with relative sparing of the pons,^43–45^ and the pons-to-midbrain ratio has high specificity and sensitivity for the diagnosis of pathogically confirmed PSP.^46^ SCP atrophy is also evident early in the disease course^47^ and has led to the development of the MR parkinsonism index for differentiation PSP-RS from other causes of parkinsonism.^48^ Atrophy of subcortical structures including the striatum, GP and thalamus has also been observed in group-level studies,^49–54^ as well as involvement of frontal lobe.^55–57^ Together, these findings are consistent with the sequence of atrophy that the EBM produces, but our study is the first in PSP-RS, to the best of our knowledge, that orders these regions relative to each other.

The placement of the medulla first in the sequence is interesting as the medulla is not widely mentioned in the PSP imaging literature. It is, however, clear that tau pathology is consistently seen in the medulla at post-mortem,^58,59^ with Kovacs *et al*.^8^ placing it at Step 2 in their pathological staging system. More recently, perhaps due to the advent of automated segmentation techniques for the brainstem, its involvement has been shown in PSP-RS using MRI.^44,45,60,61^ The early involvement of the thalamus in our EBM sequence is also supported both by pathological studies^8^ where tau pathology been shown to occur in all cases, and structural MRI studies that demonstrate atrophy: in particular the pulvinar, dorsomedial and anterior nuclei.^62,63^ In future work, it will be interesting to investigate differential involvement of the thalamic nuclei in the different PSP subtypes, and their position in the event ordering relative to downstream atrophy events.

### Patient staging

This EBM demonstrates that there is significant variability in terms of the stage of PSP-RS patients at baseline (Fig. 2) and provides an intrinsic staging mechanism by which to stratify patients more accurately in terms of their temporal position in the disease course. This is supported by the association between EBM stage and disease duration (both at all timepoints and only at baseline) in those subjects for which disease duration was recorded (Fig. 4B).

Uncertainty in the model assigned stage is dependent on the degree of overlap between the HC and PSP-RS biomarker distributions, as well as the accuracy of a given person’s biomarker measurement.^23^ Imaging biomarkers are known to be associated with a high degree of variance, some of which can be explained by different scanners used, the age and gender and variation in individual TIV. We tried to control for this by regressing these out as covariates. Linear modelling of age against predicted EBM stage for cases and controls (Supplementary Fig. 4A and B) showed no association supporting the validity of this approach.

Although the purpose of this study was to identify the sequence of regional atrophy in PSP-RS from cross-sectional data, rather than classify subjects as cases versus controls, using a threshold of Stage 2 (medulla and midbrain atrophic), the model was able to correctly classify subjects as PSP-RS versus HC with an overall categorization accuracy of 90%. This accuracy is similar to that seen in other MRI studies using simple group wise comparisons of midbrain volume between cases and controls^60^ and gives confidence that the EBM sequence is a valid representation of disease progression. This is further supported by the fact that 96% of cases either stayed at the same stage or progressed to a higher stage over a 12-month period. In addition, predicted subject EBM stage is significantly correlated (*P* < 0.01) with a validated measure of clinical disease severity (PSP rating scale), as well as disease duration (*P* < 0.01), demonstrating the clinical relevance of our MRI-based fine-grained staging system. However, unlike a clinical rating score, the EBM also provides insights into the underlying progression of brain volume changes, and given it is probabilistic, a natural way to incorporate uncertainty into the staging.

### Limitations

There are several assumptions made when building an EBM, which must be considered when interpreting our results. The EBM assumes that all patients have a broadly similar disease progression pattern with a unimodal distribution of orderings We restricted analysis to those patients with a diagnosis of PSP-RS, to try and exclude some of the heterogeneity in clinical phenotype associated with PSP pathology.^4^ Those cases included from the 4RTNI1, Davunetide and SAL/YP cohorts were diagnosed with probable PSP-RS according to the NINDS criteria, though it is possible that at least some of these cases may meet the 2017 diagnostic criteria for non-RS clinical phenotypes. In the Prospect study, 10% of PSP cases diagnosed under the NINDS criteria were relabelled as a non-RS phenotype when the 2017 MDS criteria were applied.^61^ Given the sensitivity of the EBM to sample heterogeneity, and the variation in pathology staging by phenotype,^8,9^ investigation of PSP phenotype heterogeneity using subtype and stage inference^64^ may provide finer grained patient stratification and is worth pursuing.

The EBM staging has no explicit timescale,^23^ although it can predict what stage the patient *is* within the sequence of biomarker abnormalities, it is unable in itself to extract information on the time taken to transition between states. When given longitudinal data, the model currently treats repeated measures as if they are independent i.e. from separate individuals, thus losing information on temporal covariance that could further inform on the ordering of events. Recently, a new generative model called the temporal event-based model (TEBM) has been developed^65^ to accommodate longitudinal data, which is able to learn both individual-level trajectories within the sequence of biomarker abnormalities as well as the time to transition between each event. Applied to our data set, the TEBM may provide insights into the transition times between each stage defined by this study.

Although PSP-RS has been shown to be highly correlated with underlying PSP pathology,^66^ in rare cases other pathologies such as CBD can present with PSP-RS and imaging is unable to differentiate the underlying pathology.^67^ Of the 365 PSP-RS cases selected for image processing, 24/26 (92%) of cases that came to post-mortem had PSP pathology, whereas one had GGT and the other CBD pathology (these were excluded from the analysis). Although a small sample size, this correlation between PSP-RS and underlying PSP pathology is in keeping with previous studies.^66^ In the absence of a sensitive and specific tau-PET ligand, or indeed any other biomarker, for PSP pathology, there is not an easy way around this clinical–pathological disconnect, and until such time the inclusion of patients in clinical trials based on a clinical diagnosis of PSP-RS is likely to continue.

Another limitation, although not unique to this study, is that the MRIs of different patients were acquired across a range of centres and on different scanners. It is well known that scanners can differ from each other in relation to imaging quality, signal homogeneity and image contrast which can lead to bias.^15^ Stringent visual QCs were applied to both the raw images and post-segmentation scans, the GIF algorithm bias corrects for field inhomogeneity, and we also controlled for scanner type by introducing it as a covariate in the linear regression. In addition, previous analyses on the davunetide data set (which had the highest number of different scanners) scanner type showed no significant effect on atrophy rates.^68^ Furthermore, the use of different scanners at multiple sites is a realistic scenario for clinical trials in rare diseases such as PSP, and so scanner heterogeneity combined with the large sample size in this study supports stronger generalizability of the findings.

## Conclusion

In this study, we have uncovered the *in vivo* sequence of brain atrophy in a large series of individuals with PSP-RS using a probabilistic data-driven model of brain volume changes that mirrors the recent post-mortem brain histopathology staging proposed by Kovacs *et al.*^1^ It provides an objective, *in vivo* staging system that is longitudinally consistent and correlates with clinical measures of disease severity and disease duration. This approach has potential utility to stratify PSP patients on entry into clinical trials based on disease stage, and complement existing clinical outcome measures to track disease progression.

## Supplementary Material

fcac098_Supplementary_Data

## Appendix

### 4RTNI Consortium

1. **Bradley F. Boeve**—Department of Neurology, Mayo Clinic, Rochester, MN 55905, USA.
2. **Brad C. Dickerson**—Departments of Neurology and Psychiatry, Frontotemporal Disorders Unit and Alzheimer’s Disease Research Center, Boston, MA, USA.
3. **Carmela M. Tartaglia**—Tanz Centre for Research in Neurodegenerative Diseases University of Toronto, Toronto, Canada.
4. **Irene Litvan**—Department of Neurosciences, University of California San Diego, La Jolla, CA, USA.
5. **Murray Grossman**—Department of Neurology, University of Pennsylvania, Philadelphia, PA, USA.
6. **Alex Pantelyat**—Department of Neurology. School of Medicine, Johns Hopkins University, Baltimore, MD, USA.
7. **Edward D. Huey**—Department of Psychiatry and Neurology, Columbia University, New York, NY, USA.
8. **David J. Irwin**—Penn Center for Neurodegenerative Disease Research, University of Pennsylvania School of Medicine, Philadelphia, PA, USA.
9. **Anne Fagan**—Department of Neurology, Washington University School of Medicine, St Louis, MO, USA.
10. **Suzanne L. Baker**—Molecular Biophysics and Integrated Bioimaging, Lawrence Berkeley National Laboratory, Berkeley, CA, USA.
11. **Arthur W. Toga**—Laboratory of Neuro Imaging, Stevens Neuroimaging and Informatics Institute, Keck School of Medicine of USC, University of Southern California, Los Angeles, CA, USA.

### PROSPECT Consortium

1. **Alyssa A. Costantini**, MSc—Department of Clinical and Movement Neurosciences, UCL (University College London) Queen Square Institute of Neurology, London, UK. a.costantini@ucl.ac.uk
2. **Henry Houlden**, FRCP, PhD—Department of Clinical and Movement Neurosciences, UCL (University College London) Queen Square Institute of Neurology, London, UK; Movement Disorders Centre, UCL Queen Square Institute of Neurology, London, UK; Department of Neuromuscular Diseases, UCL Queen Square Institute of Neurology, London, UK. h.houlden@ucl.ac.uk
3. **Christopher Kobylecki**, FRCP, PhD—Department of Neurology, Manchester Academic Health Science Centre, Salford Royal NHS (National Health Service) Foundation Trust, University of Manchester, Manchester, UK. Christopher.Kobylecki@srft.nhs.uk
4. **Michele T. M. Hu**, FRCP, PhD—Division of Neurology, Nuffield Department of Clinical Neurosciences, University of Oxford, Oxford, UK. michele.hu@ndcn.ox.ac.uk
5. **Nigel Leigh**, FRCP, PhD—Department of Neuroscience, Brighton and Sussex Medical School, Brighton, UK. P.Leigh@bsms.ac.uk

## Abbreviations

CBD =: corticobasal degeneration
DC =: diencephalon
EBM =: event-based model
GGT =: globular glial tauopathy
GIF =: geodesic information flow
GP =: global pallidus
HC =: healthy control
KDE =: kernel density estimation
MCMC =: Markov Chain Monte Carlo
NINDS =: National Institute of Neurological Disorders and Stroke
PSP rating scale =: progressive supranuclear palsy rating scale
PSP-RS =: progressive supranuclear palsy Richardson syndrome
QC =: quality control
ROI =: region of interest
SCP =: superior cerebellar peduncle
