# Supplementary material for: A data-driven model of brain volume changes in progressive supranuclear palsy
Source: Brain Commun. 2022 Apr 14;4(3):fcac098. doi: 10.1093/braincomms/fcac098 (PMC9118104; doi:10.1093/braincomms/fcac098)
Supplement: fcac098_Supplementary_Data [file fcac098_supplementary_data.zip › Original Submission.pdf]

OXFORD UNIVERSITY PRESS | Brain Communications

A data-driven model of brain volume changes in Progressive Supranuclear Palsy

Journal:	*Brain Communications*
Manuscript ID	BRAINCOM-2021-322
Manuscript Type:	Original Article
Date Submitted by the Author:	17-Sep-2021
Complete List of Authors:	Scotton, William; UCL Queen Square Institute of Neurology, Dementia Research Centre, Department of Neurodegenerative Disease<br>Bocchetta, Martina; UCL Queen Square Institute of Neurology, Dementia Research Centre, Department of Neurodegenerative Disease<br>Todd, Emily; UCL Queen Square Institute of Neurology, Dementia Research Centre, Department of Neurodegenerative Disease<br>Cash, David; UCL Queen Square Institute of Neurology, Dementia Research Centre, Department of Neurodegenerative Disease<br>Oxtoby, Neil; University College London Centre for Medical Image Computing, Department of Computer Science, University College London<br>VandeVrede, Lawren; UCSF, Department of Neurology, Memory and Aging Center<br>Heuer, Hilary; UCSF, Department of Neurology, Memory and Aging Center<br>Alexander, Daniel; University College London Centre for Medical Image Computing, Department of Computer Science, University College London<br>Rowe, James; University of Cambridge Department of Clinical Neurosciences, Medical Research Council Cognition and Brain Sciences Unit<br>Morris, Huw; UCL Queen Square Institute of Neurology, Department of Clinical and Movement Neuroscience<br>Boxer, Adam; UCSF, Department of Neurology, Memory and Aging Center<br>Rohrer, Jonathan; UCL Queen Square Institute of Neurology, Dementia Research Centre, Department of Neurodegenerative Disease<br>Wijeratne, Peter; University College London Centre for Medical Image Computing, Department of Computer Science, University College London
Keywords:	Progressive Supranuclear Palsy, Event-based modelling, Disease Progression, Biomarkers, Machine learning, Tauopathies

SCHOLARONE™ Manuscripts

A data-driven model of brain volume changes in Progressive Supranuclear Palsy

W. J. Scotton[1], M. Bocchetta[1], E. Todd[1], D. M. Cash[1], N. Oxtoby[2], L. VandeVrede[3], H. Heuer[3], PROSPECT Consortium, 4RTNI Consortium, D. C. Alexander[2], J.B. Rowe[4], H.R. Morris[5], A Boxer[3], J.D. Rohrer[†,1], P. A. Wijeratne[†,2]

Abstract

The most common clinical phenotype of Progressive Supranuclear Palsy (PSP) is Richardson syndrome (PSP-RS), characterised by levodopa unresponsive symmetric parkinsonism, with a vertical supranuclear gaze palsy, early falls, and cognitive impairment. There is currently no detailed understanding of the full sequence of disease pathophysiology in PSP. Determining the sequence of brain atrophy in PSP could provide important insights into the mechanisms of disease progression as well as guide patient stratification and monitoring for clinical trials. We used a probabilistic event-based model (EBM) applied to cross-sectional structural MRI scans in a large international cohort of people with PSP-RS, to determine the sequence of brain atrophy in clinically diagnosed PSP-RS. A total of 341 people with PSP-RS (of whom 255 had 12-month follow-up imaging) and 260 controls were included in the study. We used a combination of 12-month follow-up MRI scans, and a validated clinical rating score (PSP Rating scale) to demonstrate the longitudinal consistency and utility of the EBM's staging system. The EBM estimated that the earliest atrophy occurs in the brainstem and subcortical regions followed by progression caudally into the superior cerebellar peduncle and deep cerebellar nuclei, and rostrally to the cortex. The sequence of cortical atrophy progresses in an anterior to posterior direction, beginning in the frontal lobe before spreading to the temporal, parietal and finally the occipital lobe. This *in-vivo* ordering accords with the *post-mortem* neuropathological staging of PSP and was robust under bootstrap cross-validation. Using longitudinal information from 12- month follow-up scans we demonstrate that subjects consistently move to later stages over this time interval, supporting the validity of the model. In addition, predicted subject EBM stage was significantly correlated ($p<0.01$) with clinical severity (PSP Rating Scale). Our results provide new insights into the sequence of atrophy

For Review Only

progression in PSP and offer potential utility to stratify people with PSP on entry into clinical trials based on disease stage, as well as track disease progression.

Author affiliations:

†These authors contributed equally to this work

1. Dementia Research Centre, Department of Neurodegenerative Disease, UCL Queen Square Institute of Neurology, University College London, London, UK.
2. Centre for Medical Image Computing, Department of Computer Science, University College London, London, UK.
3. Department of Neurology, Memory and Aging Center, University of California, San Francisco, CA, USA.
4. Cambridge University Department of Clinical Neurosciences and Cambridge University Hospitals NHS Trust; Medical Research Council Cognition and Brain Sciences Unit, Cambridge UK.
5. Department of Clinical and Movement Neurosciences, University College London Queen Square Institute of Neurology, London, UK; Movement Disorders Centre, University College London Queen Square Institute of Neurology, London.

Correspondence to: Dr William J. Scotton

Full address: UCL Institute of Neurology, Department of Neurodegeneration, Dementia Research Centre, First Floor, 8-11 Queen Square, WC1N 3AR, London.

E-mail: w.scotton@ucl.ac.uk

Keywords: event-based model; disease progression; Progressive Supranuclear Palsy; biomarkers; machine learning.

Abbreviations: CBD = corticobasal degeneration; DC = diencephalon; EBM = event based model; GGT = globular glial tauopathy; GIF = geodesic information flow; GP = global pallidus; HC = healthy control; KDE = kernel density estimation; QC = quality control; MCMC = Markov Chain Monte Carlo; NINDS = National Institute of Neurological

For Review Only

Disorders and Stroke; PSP = Progressive Supranuclear Palsy; PSP-RS = Progressive Supranuclear Palsy Richardson Syndrome; PSP Rating Scale = Progressive Supranuclear Palsy Rating Scale; ROI = region of interest; SCP = superior cerebellar peduncle.

For Review Only

Introduction

Progressive Supranuclear Palsy (PSP) is a severe neurodegenerative condition, with an estimated prevalence of 5-7 per 100,000 and survival of just 5-7 years[1,2]. PSP pathology can present with a range of clinical phenotypes involving language, behavioural and movement abnormalities[3]. This heterogeneity in clinical presentation has been operationalised in the Movement Disorder Society 2017 PSP diagnostic criteria[4]. The most common clinical phenotype is Richardson syndrome (PSP-RS), similar to the cases first described by Steele, Richardson and Olszewski in 1963[5], and characterised by a levodopa unresponsive parkinsonian syndrome with a vertical supranuclear gaze palsy, early falls and dementia. Natural history studies of PSP-RS have shown the mean age of symptom onset is between 65 and 67 years with an average survival from disease onset of 6-7 years[2,6]. PSP pathology is characterised by insoluble aggregates of the 4-repeat (4R) isoform of the microtubule-associated protein tau in neurons and glia, predominantly in the subthalamic nucleus, globus pallidus, striatum, dentate nucleus of the cerebellum, frontal lobes and to a lesser extent in the occipital cortices[7]. The recent pathology staging system for PSP defines six sequential stages of progression, starting with the subthalamic nucleus, spreading out caudally to the cortex and rostrally to the cerebellum[8]. This has been validated in an independent cohort with increasing pathogical stage correlating with clinical severity[9].

No effective disease modifying treatment has yet been proven for PSP, despite recent successful clinical trials[10,11]. Clinical trials in PSP can be complicated by variable disease stage at trial entry, highlighting the importance of stratifying patients into homogenous cohorts based on disease stage with similar rates of disease progression. Although the PSP Rating Scale has been shown to be a good independent predictor of survival[12], and is used as the primary endpoint in clinical trials, such clinical biomarkers are only indirect measures of the biological stage of disease, and are affected by intra- and inter-rater variability, as well as fluctuation in patients' clinical state. Reliable and individualised disease progression markers are therefore required to complement clinical ratings scales[13].

Structural MRI reveals significant atrophy in the brainstem and subcortical structures in PSP-RS, with additional involvement of the subcortical structures[14]. Increased rates of atrophy in these regions can be detected over a 12-month period[15,16], offering a potential biomarker readout for clinical trials. While there are new tau PET tracers emerging that show potential

For Review Only

in the 4R tauopathies, these are not yet validated for use in the clinic setting[17,18], and in the absence of a validated tau PET tracer for PSP, structural MRI offers an indirect measure of underlying tau pathology *in vivo*. Indeed, a previous study in PSP showed that *in vivo* structural imaging reflected the independent contributions from tau burden and neurodegeneration at autopsy[19], while the link in Alzheimer's Disease is well established[20,21]. However, the order in which brain regions show evidence of increased atrophy *in vivo* is currently unknown.

One approach to estimating the sequence of atrophy progression is event-based modelling (EBM)[22], using a probabilistic data-driven generative model to infer the order in which biomarkers become abnormal. The EBM can be built from cross-sectional data by combining severity information across biomarkers and individuals without reference to a given individual's clinical status[23]. The EBM allows inference of longitudinal information about disease progression by assuming there is a monotonic progression of an individual biomarker from normal to abnormal (even if this progression is non-linear), so that in a patient cohort containing a spectrum of disease stages, more individuals will necessarily show abnormality in a biomarker that changes early in the disease course. This approach has been successfully applied to Huntington's disease[23], sporadic and familial Alzheimer's disease[24–26], Parkinson's disease[27], multiple sclerosis[28], the posterior cortical atrophy variant of Alzheimer's disease[29], and to amyotrophic lateral sclerosis[30], providing a simple and validated method to investigate temporal disease patterns and estimate individuals' disease stage. Recent work has demonstrated the clinical utility of the EBM for screening patients on entry into clinical trials, to improve cohort homogeneity and increase the power to detect a treatment effect[31].

The aim of this study was to define the progression of brain atrophy in clinically diagnosed PSP-RS by developing an EBM that takes cross-sectional structural MRI imaging as input. We hypothesised that there is a consistent sequence in which brain regions become atrophic in PSP-RS, in keeping with the recent PSP pathology staging system proposed by Kovacs et al.[8], and predicted that the image-based EBM stage would be correlated with clinical disease severity as measured by the PSP Rating Scale.

For Review Only

Materials and methods

Subjects

Data from individuals with a clinical diagnosis of possible or probable PSP-Richardson Syndrome were collected from six main sources for inclusion in the study: the 4R Tauopathy Imaging Initiative (4RTNI; ClinicalTrials.gov: NCT01804452)[16,32], the davunetide randomized control trial (DAV; ClinicalTrials.gov: NCT01056965)[33], the salsalate clinical trial (SAL; ClinicalTrials.gov: NCT02422485)[34], the young plasma clinical trial (YP; ClinicalTrials.gov: NCT02460731)[34], the PROgressive Supranuclear Palsy CorTico-Basal Syndrome Multiple System Atrophy Longitudinal Study (PROSPECT; ClinicalTrials.gov: NCT02778607), and the University College London Dementia Research Centre (UCL DRC) FTD cohort. Control data were collected from three sources: the Frontotemporal Lobar Degeneration Neuroimaging Initiative dataset (FTLDNI; http://4rtni-ftldni.ini.usc.edu/) PROSPECT, and the UCL DRC FTD Cohort. Further details on individual cohorts are included in the supplementary material, and a summary of the demographics of each cohort is included in Supp. Table 1. Appropriate ethics was applied for and approved via the relevant trial and research ethics committees. For inclusion in this study all patients had to have, as a minimum, a baseline T1-weighted volumetric MRI on a 1.5T or 3T scanner, with basic demographic data (age at time of scan, gender), and disease duration at time of the scan (time from symptom onset to MRI scan). 12-month follow-up scans, if available, were also included in the study, as were PSP Rating scale scores. Given original trial analyses failed to show any treatment effect (including no change in volumetric MRI measurements) in the davunetide[33], salsalate and young plasma trials[34], we combined data from each study's treatment and placebo groups. Longitudinal data (both 12-month follow-up MRI and PSP Rating Scale) were used for validation of the staging system produced by the baseline EBM.

Magnetic resonance imaging

Raw volumetric T1 MRI images were all processed by the same pipeline. Scans first underwent visual quality control (QC) to ensure correct acquisition and the absence of major artefacts. Next, raw images that passed QC were bias field corrected for magnetic field inhomogeneity, and the whole brain (cortical and subcortical structures) parcellated using the

For Review Only

geodesic information flow (GIF) algorithm[35]. This automatically extracts regions based on the Neuromorphometrics atlas (Neuromorphometrics, Inc.), using an atlas propagation and label fusion strategy[36,37]. Subregions of the cerebellum were then automatically extracted with GIF based on the Diedrichsen cerebellar atlas: the cerebellar lobules (I-IV, V, VI, VIIa-Crus I, VIIa-Crus II, VIIb, VIIIa, VIIIb, IX and X), the vermis and the deep nuclei (dentate, interposed and fastigial)[35,38]. The whole brainstem, medulla, pons, superior cerebellar peduncles (SCP) and midbrain were subsequently segmented using a customised version of the module available in FreeSurfer to accept the GIF parcellation as input for Freesurfer [39]. Total intracranial volume (TIV) was calculated using SPM12 v6225 (Statistical Parametric Mapping, Wellcome Trust Centre for Neuroimaging, London, UK) running under MATLAB R2012b (Math Works, Natick, MA, USA)[40]. All segmentations were visually inspected to ensure accurate segmentation.

Biomarker selection

In this study we use the term biomarker to refer to image-based regional brain volumes that show a significant difference between cases and healthy controls (two-tailed t-test of mean difference in covariate adjusted volumes). Given the focus of this study was to test the hypothesis that the sequence of atrophy in PSP-RS is in keeping with the sequence of tau pathology at post-mortem as shown by Kovacs et al.[8], nineteen regions of interest (ROI) were chosen for inclusion that most closely matched those used in their study; four brainstem (medulla, pons, superior cerebellar peduncle [SCP], and midbrain), three cerebellar (cerebellar cortex, deep nuclei and vermis), seven subcortical (thalamus, globus pallidus [GP], striatum [caudate and putamen], ventral diencephalon [DC], thalamus, hippocampus and amygdala) and five cortical (frontal, insula, temporal, parietal and occipital). Regions that had a right and left label were combined. All ROIs were controlled for the following covariates using linear regression on the control cohort: age at scan, sex, scanner type and TIV. All regions selected for inclusion showed a significant difference in covariate adjusted volumes between cases and controls (Bonferroni corrected threshold of $p < 1 \times 10^{-4}$) under a two-tailed t-test.

For Review Only

The Event Based Model

The EBM is designed to infer a data-driven, probabilistic sequence in which biomarkers become abnormal from cross-sectional data. The strengths of the EBM are firstly that it requires no *a-priori* biomarker cut-offs (thresholds) to define abnormality, secondly it requires no a priori staging and finally it can produce meaningful results using only moderately sized cross-sectional data. Its reliability with moderately sized datasets makes it ideally suited for analysing biomarkers in rare diseases such as the primary tauopathies.

The EBM is based on the assumptions of homogenous disease progression and monotonicity: that is all patients have a broadly similar disease progression pattern with a unimodal distribution of orderings, and biomarker change is unidirectional from normal to abnormal i.e. no remission. An 'event' is considered to have occurred when a biomarker (in this study an MRI derived regional volume), has an abnormal value ('atrophy') in comparison with the expected values measured in healthy controls. The model then estimates the sequence $S = S(1), s(2),...S(l)$ in which the biomarkers become abnormal where $S(1)$ is the first biomarker, and $S(l)$ is the last. Conceptually if biomarker A is usually abnormal when biomarker B is abnormal, but B is often abnormal without A, we infer that B occurs before A in the sequence.

The estimation procedure first fits a mixture model to control and patient data for each biomarker. In this study we decided to use a recent version of the EBM that incorporates a non-parametric method, kernel density estimation (KDE)[29], for estimating the mixture models. This approach has been shown to perform at a similar level to the classic EBM (that incorporates Gaussian mixture modelling) with parametric input data, while demonstrating superiority when the data are skewed[29]. The mixture model obtains models for the distribution of normal and abnormal values for each biomarker, providing likelihoods $P(x_{ij}|E_i)$ and $P(x_{ij}|\neg E_i)$ of observing the value, x_{ij}, of biomarker i for subject j, given that biomarker i has or has not become abnormal, respectively. The EBM combines these likelihoods to then calculate the likelihood of the full dataset $X = x_{ij}{:}i = 1,...,Z; j = 1,...,N$ for a given sequence, S:

$$P(X \mid S) = \prod_{j=1}^{N}\left[\sum_{k=0}^{Z}\left(P(k)\prod_{i=1}^{k}P(x_{ij}|E_i)\prod_{i=k+1}^{Z}P(x_{ij}|E_i)\right)\right]$$

For Review Only

(1)

j iterates over the number of subjects N, and i iterates over the number of events Z. $P(k)$ refers to the prior likelihood of being at stage k and in the absence of prior information is treated as uniform to impose as little information as possible on estimated orderings. The estimation procedure then searches for the characteristic ordering, $\grave{S}$, which is the sequence that maximises the likelihood of $P(X \mid S)$ in equation (1) [23]. This is found through a combination of a multiply initialized greedy ascent and Markov Chain Monte Carlo (MCMC) sampling, which samples from the posterior distribution on S, to find $\grave{S}$, which is simply the sequence with the highest (maximum) likelihood. The set of samples from the MCMC sampling also provides information on the uncertainty of the maximum likelihood sequence, which can be visualised on a positional variance diagram[22,23].

Patient staging

Once the characteristic sequence, S, has been obtained via the EBM, an individual sample X_j (a vector of all measurements across biomarkers i for a patient j), can be staged by evaluating the stage k that maximises the likelihood in equation (2) below[25]:

$$argmax_k P(X_j \mid \grave{S},k) = argmax_k P(k) \prod_{i=1}^{k} P(x_{ij}|E_i) \prod_{i=k+1}^{Z} P(x_{ij}|\neg E_i)$$

(2)

As before $P(k)$, the prior likelihood of being at stage k, is treated as uniform i.e., no a priori information on a particular stage. The EBM stage (Z), between 1 and the number of biomarkers, l, of subject j, is therefore given by the stage k that maximises equation (2). Each subject (case and control) had their EBM predicted stage calculated for their baseline MRI scan, and for those that had them, their 12-month follow-up scan.

Bootstrap cross validation of event sequence

Although the MCMC sampling gives some information on the uncertainty of the event ordering in ordering of events derived from the EBM, previous work shows it tends to underestimate this uncertainty[25]. Bootstrapping is an additional method that tends to give a

For Review Only

more liberal estimate of the uncertainty in the ordering. We performed cross-validation of the maximum likelihood sequence generated by the EBM, by re-estimating the model on 100 bootstrap samples of the original data (sampling with replacement).

Longitudinal validation

We investigated the longitudinal consistency of the staging produced by the EBM, based on the predictions that, firstly, given PSP is a progressive disease, the EBM stage should increase over time, and secondly that increasing EBM stage should be associated with increasing PSP Rating Scale score (the main clinical measure of disease severity), especially during later model stages where there is more widespread atrophy. We staged patients using the baseline EBM based on their 12-month follow-up scan (255 cases) and compared this with predicted stage based on their baseline scan. The follow-up data was processed using the same pipeline as the baseline scans to produce the same ROI biomarkers at 12-months. To test for the relationship of PSP Rating Scale score with baseline EBM stage, a linear mixed effects model was fit to the data using the lme4 package[41] in R Studio (version 1.4.1106), with EBM defined stage as the independent variable and PSP rating scale score as the dependent variable. All cases (255) had a corresponding PSP rating scale score. Subject Id was modelled as a random effect (random intercept) due to some subjects having two MRI scans at different time points. Significance was calculated using the lmerTest package[42] which applies Satterthwaite's method to estimate degrees of freedom and generate p-values for mixed models.

Data availability

Source data are not publicly available but non-commercial academic researcher requests may be made to the Chief Investigators of the six source studies, subject to data access agreements and conditions that preserve participant anonymity. The underlying event-based model code is publicly available at https://github.com/noxtoby/kde_ebm.

For Review Only

Results

Subject characteristics

Table 1 summarises the key demographic data for the cohort included in the study. 929 MRI images were processed from a total of 654 subjects: 365 with a clinical diagnosis of PSP-RS (of which 275 had 12-month follow-up scans) and 289 controls. Of the PSP-RS cases 26 (8%) had a pathological diagnosis after coming to post-mortem: 24 (92%) showed tau pathology consistent with PSP, while 2 cases had non-PSP tau pathology (one CBD and one GGT) and were therefore excluded from the analysis. After stringent quality control with visual inspection of all images for the remaining 363 cases (pre- and post- processing), 341 PSP-RS cases (of which 255 had 12-month follow-up scans) and 260 control scans were included for the analysis. Reasons for scans failing quality control included poor quality of the raw T1 image (usually due to movement artefacts) or inaccurate segmentations with the GIF or / and SPM algorithms. All cases included had a PSP rating scale score at baseline and follow-up, as well as recorded age, gender, scanner type and TIV. At baseline the PSP-RS cohort had an older average age (67.9 years, standard deviation [SD] ± 6.8) compared to healthy controls (62.8 years, $SD \pm 9.4$, $t = -7.4,\ p < 0.01$). Disease duration data (time from diagnosis to baseline visit [average years, ± SD]) was available for 87/341 cases and showed an average length of 4.1 years (SD ± 3.1). There was a higher proportion of females in the control group compared to the PSP-RS group (male / female, 112/148 vs 176/165 respectively, $\chi^2 = 4.3, p = 0.04$).

Sequence of atrophy progression

Supp. Fig. 1 shows histograms of the healthy control (HC) and covariate adjusted PSP-RS ROI biomarker distributions, with KDE mixture model fits and line showing probability of an event. These fits provide the parameters for the normal and abnormal likelihoods, $P(x_{ij}|E_i)$ and $P(x_{ij}|\neg E_i)$, respectively, that are then used to calculate the maximum likelihood sequence of the full dataset. At baseline all nineteen ROI selected for inclusion in the model showed a significantly smaller covariate adjusted volume in PSP-RS compared to controls.

The positional variance diagram in Fig. 1A shows the most likely sequence in which these regions become atrophic, as estimated by the EBM, as well as the uncertainty in this

For Review Only

sequence. The maximum likelihood sequence was estimated using PSP-RS cases only, based on the rationale that PSP is a rare disease, and it is very unlikely for our cohort of controls to have asymptomatic PSP. Indeed, it is more likely the controls would have a common disorder such as AD rather than PSP, and we did not want this to confound the sequence estimation hence the exclusion. The EBM estimated that the earliest atrophy occurs in the brainstem and subcortical regions followed by progression caudally into the superior cerebellar peduncle and deep cerebellar nuclei, and rostrally to the cortex. The sequence of cortical atrophy progresses in an anterior to posterior direction, beginning in the frontal lobe before then spreading to the temporal, parietal and finally the occipital lobe (Fig. 1C) The high colour intensity of each square and their presence predominantly on the diagonal of the positional variance diagram indicates that the model has a high degree of certainty regarding their positions in the overall sequence.

Bootstrap cross-validation

Fig. 1B shows positional variance of the maximum likelihood sequence re-estimated by bootstrapping of the data (random resampling with replacement 100 times) and refitting the model. The positional variance diagram for the bootstrapped results represents the proportion of bootstrap samples in which the event i (y axis) appears at position k (x axis) of the maximum likelihood sequence. The sequence ordering is generally preserved, though as one would expect with this more conservative estimate of uncertainty, there is increased uncertainty in the relative positions early in the sequence from stage two (midbrain) to stage 4 (ventral diencephalon), and in the middle from stage nine (striatum) to stage thirteen (pons).

Patient staging

Fig. 2 shows the proportion of subjects at each EBM defined stage (PSP-RS and HC). Patient staging results were evaluated using the maximum likelihood sequence (Fig. 1A) of regional atrophy for PSP-RS subjects as described in the Methods section. As one would expect the HC cohort is clustered at the early stages with greater than 80% at Stage 0 (i.e., no event occurred), while the PSP-RS cases are distributed more evenly across stages with the highest proportion in the middle to late stages. This suggests that the cohort of PSP cases gathered from multiple different studies were temporally heterogenous which supports the importance of accurately staging patients using objective biomarkers.

For Review Only

Using a threshold of stage 2 (medulla and midbrain atrophic) the model was able to correctly classify subjects as PSP-RS versus healthy control with an overall accuracy of 90% (with a sensitivity and specificity of 91% and 90% respectively). Although not the focus of this model the high classification accuracy provided by the EBM further demonstrates its clinical validity.

Outliers were present in both the HC and PSP-RS groups: specifically, 10 (4%) of PSP-RS cases were at Stage 0, while 14 controls were at Stage 10 or greater (5%). Visual inspection of these HCs suggested that the segmentations were accurate, but that there were non-specific covariate adjusted decreased volumes in regions including the hippocampus with relative sparing of the brainstem and subcortical structures, suggesting that these could potentially represent people with preclinical Alzheimer's disease.

Longitudinal consistency

To test the validity of the EBM we first tested the hypothesis that a valid model will produce non-decreasing disease stages for individuals from baseline to follow-up, within the bounds of model uncertainty. Fig. 3 compares each PSP-RS subject's EBM stage at baseline with their stage at 12-month follow-up (255 cases had both a baseline and 12-month follow-up scan). Overall, on this metric the EBM shows good longitudinal consistency with each subjects EBM stage generally increasing or remaining stable at 12-months follow-up: 245/255 cases (ninety-six percent) either stayed at the same stage or progressed. For these cases the average stage progression over 12 months was 1 stage. Of the ten PSP cases that reverted in stage, nine only dropped one stage while one dropped two stages.

To further validate the EBM, we modelled PSP rating scale as a function of predicted EBM stage using a linear mixed model (Fig. 4). EBM stage was modelled as a fixed effect while Subject Id was modelled as random effect due to some subjects having two MRI scans at different time points. We found a significant fixed effect of EBM stage on predicted PSP rating scale (β=1.46,95% CI 1.2-1.8, $p<0.001$) and a conditional R^2 of 0.56.

Discussion

The principal result of this study is that a probabilistic data-driven method reveals, *in vivo*, the sequence in which brain regions become atrophic in PSP-RS. We established this

For Review Only

sequence from cross-sectional data and went on to demonstrate the validity of this model longitudinally. Ninety-six percent remained in the same stage or progressed to a later stage over 12-months. The model derived staging correlated with clinical severity.

Ordering of biomarkers

The order of regional atrophy revealed by the EBM (Fig. 1) broadly mirrors the sequential spread of tau pathology in PSP proposed by Kovacs et al.[8]. The earliest atrophy in our model occurs in the brainstem and subcortical regions followed by progression caudally into the superior cerebellar peduncle and deep cerebellar nuclei, and rostrally to the cortex. The sequence of cortical atrophy progresses in an anterior to posterior direction, beginning in the frontal lobe before then spreading to the temporal, parietal and finally the occipital lobe. In the absence of external data to validate the model, we explored the generalisability and robustness of the model using bootstrap cross validation. This demonstrates that even with a more conservative estimate of uncertainty, the sequence of atrophy is largely conserved (Fig. 1B). There remains uncertainty early on between the relative positions of the midbrain, thalamus, ventral DC and SCP, in the middle between the striatum, frontal, parietal, and cingulate lobes, and the pons, and at the end of the sequence between the temporal lobe, amygdala, and hippocampus. This heterogeneity is of interest, and a motivation for future work.

It is difficult, however, to make a direct comparison between our *in-vivo* findings and *post-mortem* tau histopathology staging for two reasons: firstly, in this study we are measuring atrophy rather than tau pathology directly, and although there is evidence that atrophy on structural imaging is associated with tau pathology[19,20] it is unlikely to directly correlate with histopathological scores of tau accumulation across neuronal and glial cell populations. Secondly, two of the regions identified to have the earliest tau pathology in Kovacs' study are the subthalamic nucleus (STN) and the substantia nigra (SN), regions that are not individually segmented by the GIF algorithm used in this study. These are subsumed within the ventral diencephalon (ventral DC) segmentation in the Neuromorphometrics atlas, along with the hypothalamus. Although not specific for the STN and SN, reassuringly this region does occur early in the sequence (Fig. 1A), and after bootstrapping one can see (Fig. 1B) that after the medulla there is uncertainty as to the exact ordering of the midbrain, thalamus, and ventral DC.

For Review Only

The majority of cross-sectional imaging studies in PSP-RS, have focused on the clinical utility of structural MR imaging as a diagnostic biomarker to differentiate PSP from both PD and other atypical parkinsonian disorders[13]. These studies usually only give a group level overview of regional atrophy at baseline, as opposed to the sequence of atrophy changes that we have demonstrated in this study. Even so midbrain atrophy is commonly seen in PSP-RS at baseline, with relative sparing of the pons[43–45], and the pons to midbrain ratio has high specificity and sensitivity for the diagnosis of pathogically confirmed PSP[46]. SCP atrophy is also evident early in the disease course [47] and has led to the development of the MR Parkinsonism Index (MRPI) for differentiation PSP-RS from other causes of parkinsonism[48]. Atrophy of subcortical structures including the striatum, globus pallidus and thalamus has also been observed in group-level studies[49–54], as well as involvement of frontal lobe[55–57]. Together these findings are consistent with the sequence of atrophy that the EBM produces, but our study is the first in PSP-RS, to the best of our knowledge, that orders these regions relative to each other.

The placement of the medulla first in the sequence is interesting as the medulla is not widely mentioned in the PSP imaging literature. It is however clear that tau pathology is consistently seen in the medulla at post-mortem[58,59], with Kovacs[8] placing it at Step 2 in their pathological staging system. More recently, perhaps due to the advent of automated segmentation techniques for the brainstem, its involvement has been shown in PSP-RS using MRI[44,45,60,61]. The early involvement of the thalamus in our EBM sequence is also supported both by pathological studies[8] where tau pathology been shown to occur in all cases, and structural MRI studies that demonstrate atrophy: in particular the pulvinar, dorsomedial, and anterior nuclei[62,63]. In future work it will be interesting to investigate differential involvement of the thalamic nuclei in the different PSP subtypes, and their position in the event ordering relative to downstream atrophy events.

Patient staging

This EBM demonstrates that there is significant heterogeneity in terms of the stage of PSP-RS patients at baseline (Fig. 2) and provides an intrinsic staging mechanism by which to stratify patients more accurately in terms of their temporal position in the disease course. Uncertainty in the model assigned stage is dependent on the degree of overlap between the HC and PSP-RS biomarker distributions, as well as the accuracy of a given person's

For Review Only

biomarker measurement[23]. Imaging biomarkers are known to be associated with a high degree of variance, some of which can be explained by different scanners used, the age and gender, and variation in individual TIV. We tried to control for this by regressing these out as covariates.

Although the purpose of this study was to identify the sequence of regional atrophy in PSP-RS from cross-sectional data, rather than classify subjects as cases versus controls, using a threshold of stage 2 (medulla and midbrain atrophic) the model was able to correctly classify subjects as PSP-RS versus healthy control with an overall categorisation accuracy of 90%. This accuracy is similar to that seen in other MRI studies using simple group wise comparisons of midbrain volume between cases and controls[60] and gives confidence that the EBM sequence is a valid representation of disease progression. This is further supported by the fact that ninety-six percent of cases either stayed at the same stage or progressed to a higher stage over a 12-month period. In addition, predicted subject EBM stage is significantly correlated ($p<0.01$) with a validated measure of clinical disease severity (PSP Rating Scale), demonstrating the clinical relevance of our MRI-based fine-grained staging system. However, unlike a clinical rating score, the EBM also provides insights into the underlying progression of brain volume changes, and given it is probabilistic, a natural way to incorporate uncertainty into the staging.

Limitations

There are several assumptions made when building an EBM, which must be considered when interpreting our results. The EBM assumes that all patients have a broadly similar disease progression pattern with a unimodal distribution of orderings We restricted analysis to those patients with a diagnosis of PSP-RS, to try and exclude some of the heterogeneity in clinical phenotype associated with PSP pathology[4]. Those cases included from the 4RTNI1, Davunetide and SAL / YP cohorts were diagnosed with probable PSP-RS according to the NINDS criteria, though it is possible that at least some of these cases may meet the 2017 diagnostic criteria for non-RS clinical phenotypes. In the Prospect study 10% of PSP cases diagnosed under the NINDS criteria were relabelled as a non-RS phenotype when the 2017 MDS criteria were applied[61]. Given the sensitivity of the EBM to sample heterogeneity, and the variation in pathology staging by phenotype[8,9], investigation of PSP phenotype

For Review Only

heterogeneity using subtype and stage inference[64] may provide finer grained patient stratification and is worth pursuing.

The EBM staging has no explicit timescale[23], although it can predict what stage the patient *is* within the sequence of biomarker abnormalities, it is unable in itself to extract information on the time taken to transition between states. When given longitudinal data the model currently treats repeated measures as if they are independent i.e. from separate individuals, thus losing information on temporal covariance that could further inform on the ordering of events. Recently, a new generative model called the Temporal Event-Based Model (TEBM) has been developed[65] to accommodate longitudinal data, which is able to learn both individual-level trajectories within the sequence of biomarker abnormalities as well as the time to transition between each event. Applied to our dataset the TEBM may provide insights into the transition times between each stage defined by this study.

Although PSP-RS has been shown to be highly correlated with underlying PSP pathology[66], in rare cases other pathologies such as CBD can present with PSP-RS and imaging is unable to differentiate the underlying pathology[67]. Of the 365 PSP-RS cases selected for image processing, 24/26 (ninety-two percent) of cases that came to post-mortem had PSP pathology, while one had GGT and the other CBD pathology (these were excluded from the analysis). Although a small sample size this correlation between PSP-RS and underlying PSP pathology is in keeping with previous studies[66]. In the absence of a sensitive and specific tau-PET ligand, or indeed any other biomarker, for PSP pathology, there is not an easy way around this clinic-pathological disconnect, and until such time the inclusion of patients in clinical trials based on a clinical diagnosis of PSP-RS is likely to continue.

Another limitation, though not unique to this study, is that the MRIs of different patients were acquired across a range of centres and on different scanners. It is well known that scanners can differ from each other in relation to imaging quality, signal homogeneity and image contrast which can lead to bias[15]. Stringent visual quality controls were applied to both the raw images and post segmentation scans, the GIF algorithm bias corrects for field inhomogeneity, and we also controlled for scanner type by introducing it as a covariate in the linear regression. In addition, previous analyses on the davunetide dataset (which had the highest number of different scanners) scanner type showed no significant effect on atrophy rates[68]. Furthermore, the use of different scanners at multiple sites is a realistic scenario for

For Review Only

clinical trials in rare diseases such as PSP, and so scanner heterogeneity combined with the large sample size in this study supports stronger generalisability of the findings.

Conclusion

In this study we have uncovered the *in-vivo* sequence of brain atrophy in a large series of individuals with PSP-RS using a probabilistic data-driven model of brain volume changes, that mirrors the recent *post-mortem* brain histopathology staging proposed by Kovacs et al.[1] It provides an objective, *in-vivo* staging system that is longitudinally consistent and correlates with clinical measures of disease severity. This approach has potential utility to stratify PSP patients on entry into clinical trials based on disease stage, and complement existing clinical outcome measures to track disease progression

For Review Only

Acknowledgments

Part of the data used in the preparation of this manuscript were obtained from the Progressive Supranuclear Palsy-Cortico-Basal Syndrome- Multiple System Atrophy (PROSPECT) study, a UK-wide longitudinal study of patients with atypical parkinsonian syndromes (Queen Square Research Ethics Committee 14/LO/1575). Part of the data used in the preparation of this manuscript were obtained from the 4-Repeat Neuroimaging Initiative (4RTNI) database and the Frontotemporal Lobar Degeneration Neuroimaging Initiative (FTLDNI) (http://4rtni-ftldni.ini.usc.edu/). 4RTNI was launched in early 2011 and is funded through the National Institute of Aging and The Tau Research Consortium. The primary goal of 4RTNI is to identify neuroimaging and biomarker indicators for disease progression in the 4-repeat tauopathy neurodegenerative diseases, progressive supranuclear palsy (PSP) and corticobasal degeneration (CBD). FTLDNI is also founded through the National Institute of Aging and started in 2010. The primary goals of FTLDNI are to identify neuroimaging modalities and methods of analysis for tracking frontotemporal lobar degeneration (FTLD) and to assess the value of imaging versus other biomarkers in diagnostic roles. The Principal Investigator of 4RTNI is Dr. Adam Boxer, MD, PhD, at the University of California, San Francisco. The data is the result of collaborative efforts at four sites in North America. For more information on 4RTNI, please visit: http://memory.ucsf.edu/research/studies/4rtni-2. The Principal Investigator of NIFD is Dr. Howard Rosen, MD at the University of California, San Francisco. The data is the result of collaborative efforts at three sites in North America. For up-to-date information on participation and protocol, please visit: http://memory.ucsf.edu/research/studies/nifd

Funding

We thank the research participants for their contribution to the study. The Dementia Research Centre is supported by Alzheimer's Research UK, Alzheimer's Society, Brain Research UK, and The Wolfson Foundation. This work was supported by the NIHR UCL/H Biomedical Research Centre, the Leonard Wolfson Experimental Neurology Centre (LWENC) Clinical Research Facility, and the UK Dementia Research Institute, which receives its funding from UK DRI Ltd, funded by the UK Medical Research Council, Alzheimer's Society and Alzheimer's Research UK. The PROSPECT study is funded by the PSP Association and CBD Solutions. The 4-Repeat Tauopathy Neuroimaging Initiative (4RTNI) and FTLDNI are

funded by the National Institutes of Health Grant R01 AG038791) and through generous contributions from the Tau Research Consortium. Both are coordinated through the University of California, San Francisco, Memory and Aging Center. 4RTNI data are disseminated by the Laboratory for Neuro Imaging at the University of Southern California.

WJS is supported by a Wellcome Trust Clinical PhD fellowship (220582/Z/20/Z). MB is supported by a Fellowship award from the Alzheimer's Society, UK (AS-JF-19a-004-517) and the UK Dementia Research Institute. NPO is a UKRI Future Leaders Fellow (MR/S03546X/1). DCA is supported by the EPSRC (EP/M020533/1); MRC (MR/T046422/1); Wellcome Trust (UNS113739). DMC is supported by the UK Dementia Research Institute, as well as Alzheimer's Research UK (ARUK-PG2017-1946) and the UCL/UCLH NIHR Biomedical Research Centre. HRM is supported by Parkinson's UK, Cure Parkinson's Trust, PSP Association, CBD Solutions, Drake Foundation, Medical Research Council, and the Michael J Fox Foundation. HH is supported by NIH (R01AG038791, U19AG063911). LVV is supported by NIH (R01AG038791). JBR is supported by the Wellcome Trust (220258); NIHR Cambridge Biomedical Research Centre (BRC-1215-20014); PSP Association; Evelyn Trust; Medical Research Council (SUAG051 R101400). ALB is supported by NIH U19AG063911, R01AG038791, R01AG073482, U24AG057437, the Rainwater Charitable Foundation, the Bluefield Project to Cure FTD, the Alzheimer's Association and the Association for Frontotemporal Degeneration. JDR is supported by the Miriam Marks Brain Research UK Senior Fellowship and has received funding from an MRC Clinician Scientist Fellowship (MR/M008525/1) and the NIHR Rare Disease Translational Research Collaboration (BRC149/NS/MH). PAW is supported by an MRC Skills Development Fellowship (MR/T027770/1).

For Review Only

Competing interests

The authors report no competing interests.

Supplementary material

'Supplementary material is available at *Brain* online'

Appendix

4RTNI Consortium

1. **Bradley F. Boeve** - Department of Neurology, Mayo Clinic, Rochester, MN 55905, USA
2. **Brad C. Dickerson** - Departments of Neurology and Psychiatry, Frontotemporal Disorders Unit and Alzheimer's Disease Research Center, Boston Massachusetts USA.
3. **Carmela M. Tartaglia** - Tanz Centre for Research in Neurodegenerative Diseases University of Toronto Toronto Canada.
4. **Irene Litvan** - Department of Neurosciences, University of California San Diego, La Jolla, California, USA.
5. **Murray Grossman** - Department of Neurology, University of Pennsylvania, Philadelphia, USA.
6. **Alex Pantelyat** – Department of Neurology. School of Medicine, Johns Hopkins University, Baltimore, MD, USA.
7. **Edward D. Huey** - Department of Psychiatry and Neurology, Columbia University, New York, New York, USA.
8. **David J. Irwin** - Penn Center for Neurodegenerative Disease Research, University of Pennsylvania School of Medicine, Philadelphia, PA, USA.
9. **Anne Fagan** - Department of Neurology, Washington University School of Medicine, St Louis, MO, USA.
10. **Suzanne L. Baker** - Molecular Biophysics and Integrated Bioimaging, Lawrence Berkeley National Laboratory, Berkeley, CA, USA.
11. **Arthur W. Toga** - Laboratory of Neuro Imaging, Stevens Neuroimaging and Informatics Institute, Keck School of Medicine of USC, University of Southern California, Los Angeles, CA, United States.

PROSPECT Consortium

1. **Alyssa A. Costantini**, MSc - Department of Clinical and Movement Neurosciences, UCL (University College London) Queen Square Institute of Neurology, London, United Kingdom. a.costantini@ucl.ac.uk.

For Review Only

2. **Henry Houlden**, FRCP, PhD - Department of Clinical and Movement Neurosciences, UCL (University College London) Queen Square Institute of Neurology, London, United Kingdom; Movement Disorders Centre, UCL Queen Square Institute of Neurology, London, United Kingdom; Department of Neuromuscular Diseases, UCL Queen Square Institute of Neurology, London, United Kingdom. h.houlden@ucl.ac.uk
3. **Christopher Kobylecki**, FRCP, PhD - Department of Neurology, Manchester Academic Health Science Centre, Salford Royal NHS (National Health Service) Foundation Trust, University of Manchester, Manchester, United Kingdom. Christopher.Kobylecki@srft.nhs.uk
4. **Michele T. M. Hu**, FRCP, PhD - Division of Neurology, Nuffield Department of Clinical Neurosciences, University of Oxford, Oxford, United Kingdom michele.hu@ndcn.ox.ac.uk
5. **Nigel Leigh**, FRCP, PhD - Department of Neuroscience, Brighton and Sussex Medical School, Brighton, United Kingdom. P.Leigh@bsms.ac.uk

For Review Only

References

1. Schrag A, Ben-Shlomo Y, Quinn NP. Prevalence of progressive supranuclear palsy and multiple system atrophy: A cross-sectional study. *Lancet*. 1999;354(9192):1771-1775. doi:10.1016/S0140-6736(99)04137-9

2. Coyle-Gilchrist ITS, Dick KM, Patterson K, et al. Prevalence, characteristics, and survival of frontotemporal lobar degeneration syndromes. *Neurology*. 2016;86(18):1736-1743. doi:10.1212/WNL.0000000000002638

3. Boxer AL, Yu JT, Golbe LI, Litvan I, Lang AE, Höglinger GU. Advances in progressive supranuclear palsy: new diagnostic criteria, biomarkers, and therapeutic approaches. *Lancet Neurol*. 2017;16(7):552-563. doi:10.1016/S1474-4422(17)30157-6

4. Höglinger GU, Respondek G, Stamelou M, et al. Clinical diagnosis of progressive supranuclear palsy: The movement disorder society criteria. *Mov Disord*. 2017;32(6):853-864. doi:10.1002/mds.26987

5. Steele JC, Richardson JC, Olszewski J. Progressive Supranuclear Palsy: A Heterogeneous Degeneration Involving the Brain Stem, Basal Ganglia and Cerebellum With Vertical Gaze and Pseudobulbar Palsy, Nuchal Dystonia and Dementia. *Arch Neurol*. 1964;10(4):333-359. doi:10.1001/archneur.1964.00460160003001

6. Nath U, Ben-Shlomo Y, Thomson RG, Lees AJ, Burn DJ. Clinical features and natural history of progressive supranuclear palsy: A clinical cohort study. *Neurology*. 2003;60(6):910-916. doi:10.1212/01.WNL.0000052991.70149.68

7. Stamelou M, Respondek G, Giagkou N, Whitwell JL, Kovacs GG, Höglinger GU. Evolving concepts in progressive supranuclear palsy and other 4-repeat tauopathies. *Nat Rev Neurol*. 2021;0123456789. doi:10.1038/s41582-021-00541-5

8. Kovacs GG, Lukic MJ, Irwin DJ, et al. Distribution patterns of tau pathology in progressive supranuclear palsy. *Acta Neuropathol*. 2020;140(2):99-119. doi:10.1007/s00401-020-02158-2

For Review Only

9. Briggs M, Allinson KSJ, Malpetti M, Spillantini MG, Rowe JB, Kaalund SS. Validation of the new pathology staging system for progressive supranuclear palsy. *Acta Neuropathol*. 2021;141(5):787-789. doi:10.1007/s00401-021-02298-z

10. Höglinger GU, Litvan I, Mendonca N, et al. Safety and efficacy of tilavonemab in progressive supranuclear palsy: a phase 2, randomised, placebo-controlled trial. *Lancet Neurol*. 2021;20(3):182-192. doi:10.1016/S1474-4422(20)30489-0

11. Dam T, Boxer AL, Golbe LI, et al. Safety and efficacy of anti-tau monoclonal antibody gosuranemab in progressive supranuclear palsy: a phase 2, randomized, placebo-controlled trial. *Nat Med*. 2021;27(8):1451-1457. doi:10.1038/s41591-021-01455-x

12. Golbe LI, Ohman-Strickland PA. A clinical rating scale for progressive supranuclear palsy. *Brain*. 2007;130(6):1552-1565. doi:10.1093/brain/awm032

13. van Eimeren T, Antonini A, Berg D, et al. Neuroimaging biomarkers for clinical trials in atypical parkinsonian disorders: Proposal for a Neuroimaging Biomarker Utility System. *Alzheimer's Dement Diagnosis, Assess Dis Monit*. 2019;11:301-309. doi:10.1016/j.dadm.2019.01.011

14. Whitwell JL, Höglinger GU, Antonini A, et al. Radiological biomarkers for diagnosis in PSP: Where are we and where do we need to be? *Mov Disord*. 2017;32(7):955-971. doi:10.1002/mds.27038

15. Höglinger GU, Schöpe J, Stamelou M, et al. Longitudinal magnetic resonance imaging in progressive supranuclear palsy: A new combined score for clinical trials. *Mov Disord*. 2017;32(6):842-852. doi:10.1002/mds.26973

16. Dutt S, Binney RJ, Heuer HW, et al. Progression of brain atrophy in PSP and CBS over 6 months and 1 year. *Neurology*. 2016;87(19):2016-2025. doi:10.1212/WNL.0000000000003305

17. Tagai K, Ono M, Kubota M, et al. High-Contrast In Vivo Imaging of Tau Pathologies in Alzheimer's and Non-Alzheimer's Disease Tauopathies. *Neuron*. 2021;109(1):42-

For Review Only

58.e8. doi:10.1016/j.neuron.2020.09.042

18. Brendel M, Barthel H, Eimeren T Van, et al. Assessment of 18 F-PI-2620 as a Biomarker in Progressive Supranuclear Palsy. 2020:1-12. doi:10.1001/jamaneurol.2020.2526

19. Spina S, Brown JA, Deng J, et al. Neuropathological correlates of structural and functional imaging biomarkers in 4-repeat tauopathies. *Brain*. 2019;142(7):2068-2081. doi:10.1093/brain/awz122

20. Joie R La, Visani A V., Baker SL, et al. Prospective longitudinal atrophy in Alzheimer's disease correlates with the intensity and topography of baseline tau-PET. *Sci Transl Med*. 2020;12(524):5732. doi:10.1126/scitranslmed.aau5732

21. Ossenkoppele R, Lyoo CH, Sudre CH, et al. Distinct tau PET patterns in atrophy-defined subtypes of Alzheimer's disease. *Alzheimer's Dement*. 2020;16(2):335-344. doi:10.1016/j.jalz.2019.08.201

22. Fonteijn HM, Modat M, Clarkson MJ, et al. An event-based model for disease progression and its application in familial Alzheimer's disease and Huntington's disease. *Neuroimage*. 2012;60(3):1880-1889. doi:10.1016/j.neuroimage.2012.01.062

23. Wijeratne PA, Young AL, Oxtoby NP, et al. An image-based model of brain volume biomarker changes in Huntington's disease. *Ann Clin Transl Neurol*. 2018;5(5):570-582. doi:10.1002/acn3.558

24. Oxtoby NP, Young AL, Cash DM, et al. Data-driven models of dominantly-inherited Alzheimer's disease progression. *Brain*. 2018;141(5):1529-1544. doi:10.1093/brain/awy050

25. Young AL, Oxtoby NP, Daga P, et al. A data-driven model of biomarker changes in sporadic Alzheimer's disease. *Brain*. 2014;137(9):2564-2577. doi:10.1093/brain/awu176

26. O'Connor A, Weston PSJ, Pavisic IM, et al. Quantitative detection and staging of

For Review Only

presymptomatic cognitive decline in familial Alzheimer's disease: A retrospective cohort analysis. *Alzheimer's Res Ther*. 2020;12(1):1-9. doi:10.1186/s13195-020-00695-2

27. Oxtoby NP, Leyland L-A, Aksman LM, et al. Sequence of clinical and neurodegeneration events in Parkinson's disease progression. *Brain*. February 2021. doi:10.1093/brain/awaa461

28. Eshaghi A, Marinescu R V., Young AL, et al. Progression of regional grey matter atrophy in multiple sclerosis. *Brain*. 2018;141(6):1665-1677. doi:10.1093/brain/awy088

29. Firth NC, Primativo S, Brotherhood E, et al. Sequences of cognitive decline in typical Alzheimer's disease and posterior cortical atrophy estimated using a novel event-based model of disease progression. *Alzheimer's Dement*. 2020;16(7):965-973. doi:10.1002/alz.12083

30. Gabel MC, Broad RJ, Young AL, et al. Evolution of white matter damage in amyotrophic lateral sclerosis. *Ann Clin Transl Neurol*. 2020;7(5):722-732. doi:10.1002/acn3.51035

31. Oxtoby NP, Shand C, Cash DM, Alexander DC, Barkhof F. Targeted screening for Alzheimer's disease clinical trials using data-driven disease progression models. *medRxiv*. February 2021:2021.01.29.21250773. doi:10.1101/2021.01.29.21250773

32. Zhang Y, Walter R, Ng P, et al. Progression of microstructural degeneration in progressive supranuclear palsy and corticobasal syndrome: A longitudinal diffusion tensor imaging study. *PLoS One*. 2016;11(6):1-13. doi:10.1371/journal.pone.0157218

33. Boxer AL, Lang AE, Grossman M, et al. Davunetide in patients with progressive supranuclear palsy: A randomised, double-blind, placebo-controlled phase 2/3 trial. *Lancet Neurol*. 2014;13(7):676-685. doi:10.1016/S1474-4422(14)70088-2

34. VandeVrede L, Dale ML, Fields S, et al. Open-Label Phase 1 Futility Studies of Salsalate and Young Plasma in Progressive Supranuclear Palsy. *Mov Disord Clin*

For Review Only

Pract. 2020;7(4):440-447. doi:10.1002/mdc3.12940

35. Cardoso MJ, Modat M, Wolz R, et al. Geodesic Information Flows: Spatially-Variant Graphs and Their Application to Segmentation and Fusion. *IEEE Trans Med Imaging*. 2015;34(9):1976-1988. doi:10.1109/TMI.2015.2418298

36. Johnson EB, Gregory S, Johnson HJ, et al. Recommendations for the use of automated gray matter segmentation tools: Evidence from Huntington's disease. *Front Neurol*. 2017;8(OCT):519. doi:10.3389/fneur.2017.00519

37. Perlaki G, Horvath R, Nagy SA, et al. Comparison of accuracy between FSL's FIRST and Freesurfer for caudate nucleus and putamen segmentation. *Sci Rep*. 2017;7(1):1-9. doi:10.1038/s41598-017-02584-5

38. Diedrichsen J, Balsters JH, Flavell J, Cussans E, Ramnani N. A probabilistic MR atlas of the human cerebellum. *Neuroimage*. 2009;46(1):39-46. doi:10.1016/j.neuroimage.2009.01.045

39. Iglesias JE, Van Leemput K, Bhatt P, et al. Bayesian segmentation of brainstem structures in MRI. *Neuroimage*. 2015;113:184-195. doi:10.1016/j.neuroimage.2015.02.065

40. Malone IB, Leung KK, Clegg S, et al. Accurate automatic estimation of total intracranial volume: A nuisance variable with less nuisance. *Neuroimage*. 2015;104:366-372. doi:10.1016/j.neuroimage.2014.09.034

41. Bates D, Mächler M, Bolker B, Walker S. Fitting Linear Mixed-Effects Models Using lme4. *J Stat Softw*. 2015;67(1):1-48. doi:10.18637/JSS.V067.I01

42. Kuznetsova A, Brockhoff PB, Christensen RHB. lmerTest Package: Tests in Linear Mixed Effects Models. *J Stat Softw*. 2017;82(1):1-26. doi:10.18637/JSS.V082.I13

43. Cosottini M, Ceravolo R, Faggioni L, et al. Assessment of midbrain atrophy in patients with progressive supranuclear palsy with routine magnetic resonance imaging. *Acta Neurol Scand*. 2007;116(1):37-42. doi:10.1111/j.1600-0404.2006.00767.x

For Review Only

44. Bocchetta M, Iglesias JE, Chelban V, et al. Automated brainstem segmentation detects differential involvement in atypical parkinsonian syndromes. *J Mov Disord*. 2020;13(1):39-46. doi:10.14802/jmd.19030

45. Sjöström H, Granberg T, Hashim F, Westman E, Svenningsson P. Automated brainstem volumetry can aid in the diagnostics of parkinsonian disorders. *Park Relat Disord*. 2020;79:18-25. doi:10.1016/j.parkreldis.2020.08.004

46. Massey LA, Jäger HR, Paviour DC, et al. The midbrain to pons ratio. *Neurology*. 2013;80:1856-1861. https://www.ncbi.nlm.nih.gov/pmc/articles/PMC3908351/pdf/WNL205033.pdf.

47. Paviour DC, Price SL, Stevens JM, Lees AJ, Fox NC. Quantitative MRI measurement of superior cerebellar peduncle in progressive supranuclear palsy. *Neurology*. 2005;64(4):675-679. doi:10.1212/01.WNL.0000151854.85743.C7

48. Quattrone A, Morelli M, Williams DR, et al. MR parkinsonism index predicts vertical supranuclear gaze palsy in patients with PSP-parkinsonism. *Neurology*. 2016;87(12):1266-1273. doi:10.1212/WNL.0000000000003125

49. Massey LA, Micallef C, Paviour DC, et al. Conventional magnetic resonance imaging in confirmed progressive supranuclear palsy and multiple system atrophy. *Mov Disord*. 2012;27(14):1754-1762. doi:10.1002/mds.24968

50. Messina D, Cerasa A, Condino F, et al. Patterns of brain atrophy in Parkinson's disease, progressive supranuclear palsy and multiple system atrophy. *Park Relat Disord*. 2011;17(3):172-176. doi:10.1016/j.parkreldis.2010.12.010

51. Josephs KA, Whitwell JL, Dickson DW, et al. Voxel-based morphometry in autopsy proven PSP and CBD. *Neurobiol Aging*. 2008;29(2):280-289. doi:10.1016/j.neurobiolaging.2006.09.019

52. Whitwell JL, Avula R, Master A, et al. Disrupted thalamocortical connectivity in PSP: A resting-state fMRI, DTI, and VBM study. *Park Relat Disord*. 2011;17(8):599-605. doi:10.1016/j.parkreldis.2011.05.013

For Review Only

53. Saini J, Bagepally BS, Sandhya M, et al. Subcortical structures in progressive supranuclear palsy: Vertex-based analysis. *Eur J Neurol*. 2013;20(3):493-501. doi:10.1111/j.1468-1331.2012.03884.x

54. Looi JCL, Macfarlane MD, Walterfang M, et al. Morphometric analysis of subcortical structures in progressive supranuclear palsy: In vivo evidence of neostriatal and mesencephalic atrophy. *Psychiatry Res - Neuroimaging*. 2011;194(2):163-175. doi:10.1016/j.pscychresns.2011.07.013

55. Brenneis C, Seppi K, Schocke M, Benke T, Wenning GK, Poewe W. Voxel based morphometry reveals a distinct pattern of frontal atrophy in progressive supranuclear palsy. *J Neurol Neurosurg Psychiatry*. 2004;75(2):246-249. doi:10.1136/jnnp.2003.015297

56. Cordato NJ, Pantelis C, Halliday GM, et al. Frontal atrophy correlates with behavioural changes in progressive supranuclear palsy. *Brain*. 2002;125(4):789-800. doi:10.1093/brain/awf082

57. Josephs KA, Whitwell JL, Eggers SD, Senjem ML, Jack CR. Gray matter correlates of behavioral severity in progressive supranuclear palsy. *Mov Disord*. 2011;26(3):493-498. doi:10.1002/mds.23471

58. Hauw JJ, Daniel SE, Dickson D, et al. Preliminary NINDS neuropathologic criteria for steele-richardson-olszewski syndrome(progressive supranuclear palsy). *Neurology*. 1994;44(11):2015-2019. doi:10.1212/wnl.44.11.2015

59. Dickson DW, Ahmed Z, Algom AA, Tsuboi Y, Josephs KA. Neuropathology of variants of progressive supranuclear palsy. *Curr Opin Neurol*. 2010;23(4):394-400. doi:10.1097/WCO.0b013e32833be924

60. Pyatigorskaya N, Yahia-Cherif L, Gaurav R, et al. Multimodal Magnetic Resonance Imaging Quantification of Brain Changes in Progressive Supranuclear Palsy. *Mov Disord*. 2020;35(1):161-170. doi:10.1002/mds.27877

61. Jabbari E, Holland N, Chelban V, et al. Diagnosis Across the Spectrum of Progressive

For Review Only

Supranuclear Palsy and Corticobasal Syndrome. *JAMA Neurol*. 2020;77(3):377-387. doi:10.1001/jamaneurol.2019.4347

62. Padovani A, Borroni B, Brambati SM, et al. Diffusion tensor imaging and voxel based morphometry study in early progressive supranuclear palsy. *J Neurol Neurosurg Psychiatry*. 2006;77(4):457-463. doi:10.1136/jnnp.2005.075713

63. Bocchetta M, Iglesias JE, Neason M, Cash DM, Warren JD, Rohrer JD. Thalamic nuclei in frontotemporal dementia: Mediodorsal nucleus involvement is universal but pulvinar atrophy is unique to C9orf72. *Hum Brain Mapp*. 2020;41(4):1006-1016. doi:10.1002/hbm.24856

64. Young AL, Marinescu R V., Oxtoby NP, et al. Uncovering the heterogeneity and temporal complexity of neurodegenerative diseases with Subtype and Stage Inference. *Nat Commun*. 2018;9(1). doi:10.1038/s41467-018-05892-0

65. Wijeratne PA, Alexander DC. Learning transition times in event sequences: the Event-Based Hidden Markov Model of disease progression. 2020:1-8. http://arxiv.org/abs/2011.01023.

66. Osaki Y, Ben-Shlomo Y, Lees AJ, et al. Accuracy of clinical diagnosis of progressive supranuclear palsy. *Mov Disord*. 2004;19(2):181-189. doi:10.1002/mds.10680

67. Whitwell JL, Jack CR, Parisi JE, et al. Midbrain atrophy is not a biomarker of progressive supranuclear palsy pathology. *Eur J Neurol*. 2013;20(10):1417-1422. doi:10.1111/ene.12212

68. Tsai RM, Lobach I, Bang J, et al. Clinical correlates of longitudinal brain atrophy in progressive supranuclear palsy. *Park Relat Disord*. 2016;28:29-35. doi:10.1016/j.parkreldis.2016.04.006

For Review Only

Figure legends

Figure 1: Sequence of atrophy progression in PSP Richardson Syndrome. (A) Regional volume biomarker positional variance diagram showing the sequence of atrophy progression in PSP-RS. **(B)** Re-estimation of positional variance after cross-validation of the maximum likelihood event sequence by bootstrap resampling. For figures **(A)** and **(B)** the vertical ordering on the y-axis (from top to bottom) shows the maximum likelihood sequence estimated by the EBM (earliest to latest event). Colour intensity of the squares represents the posterior confidence in each biomarker's position in the sequence, from either **(A)** MCMC samples of the posterior or **(B)** bootstrapping. SCP = superior cerebellar peduncle, Ventral DC = ventral diencephalon. Note that because these volumes are covariate adjusted the control distribution will be centred at zero. **(C)** Graphic representation of the event sequence with relevant region transitioning from healthy (grey) to unhealthy (coloured). Dark red = first regions to atrophy, Light yellow = last regions to atrophy. Created with BioRender.com.

Figure 2: Histogram of event-based model staging results for PSP-RS. Healthy controls in blue and PSP-RS cases in orange. Each bar represents the proportion of patients in each category at each EBM stage. Each EBM stage on x-axis represents the occurrence of a new biomarker transition event. Stage 0 corresponds to no events having occurred and Stage 19 corresponds to all events having occurred. Events are ordered by the maximum likelihood sequence for the whole PSP-RS population as shown in Fig. 1A.

Figure 3: Longitudinal consistency of baseline EBM. Scatter plot showing predicted stage at baseline (x-axis) versus predicted stage at 12 months (y-axis) for those PSP-RS subjects with a follow-up scan (n = 255). The area of a circle is weighted by the number of subjects at each point.

Figure 4: Association between predicted EBM stage and PSP Rating Scale score. PSP Rating Scale score versus EBM stage with line representing the linear fixed effect model fit to all subjects, and 95% confidence intervals. Subject Id was modelled as a random effect due to some subjects having two MRI scans at different time points.

For Review Only

Supplementary Figure 1: **Kernel Density Estimation (KDE) mixture models.** Healthy controls (blue) and PSP-RS (orange) volume biomarker distributions, and corresponding KDE mixture model fits. The purple line represents the probability that an event has occurred $P(x_{ij}|E_i)$. Note that the volumes are covariate corrected.

For Review Only

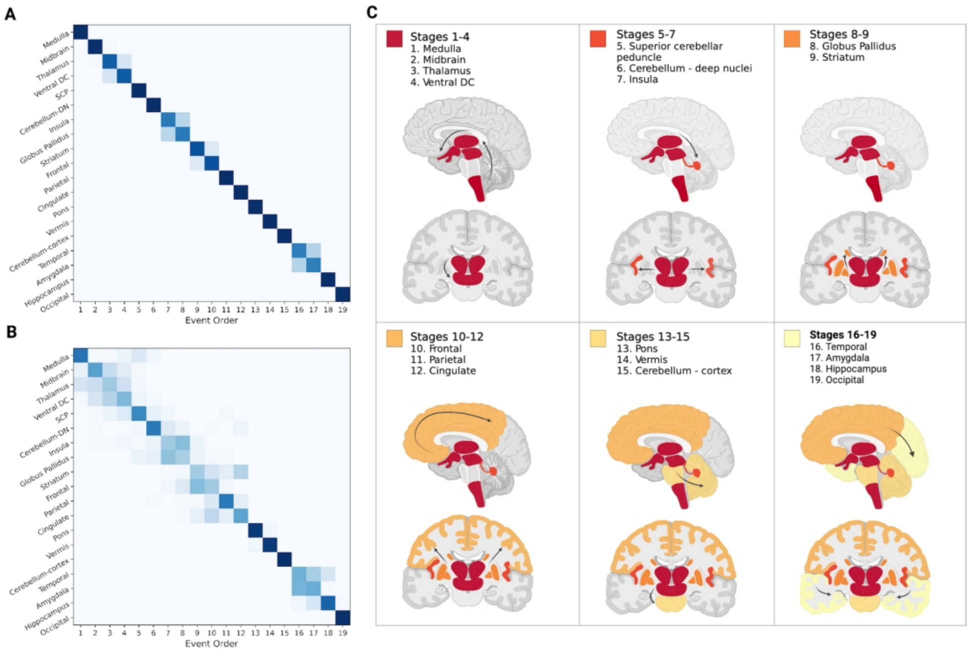

Figure 1: Sequence of atrophy progression in PSP Richardson Syndrome. (A) Regional volume biomarker positional variance diagram showing the sequence of atrophy progression in PSP-RS. (B) Re-estimation of positional variance after cross-validation of the maximum likelihood event sequence by bootstrap resampling. For figures (A) and (B) the vertical ordering on the y-axis (from top to bottom) shows the maximum likelihood sequence estimated by the EBM (earliest to latest event). Colour intensity of the squares represents the posterior confidence in each biomarker's position in the sequence, from either (A) MCMC samples of the posterior or (B) bootstrapping. SCP = superior cerebellar peduncle, Ventral DC = ventral diencephalon. Note that because these volumes are covariate adjusted the control distribution will be centred at zero. (C) Graphic representation of the event sequence with relevant region transitioning from healthy (grey) to unhealthy (coloured). Dark red = first regions to atrophy, Light yellow = last regions to atrophy. Created with BioRender.com.

168x111mm (300 x 300 DPI)

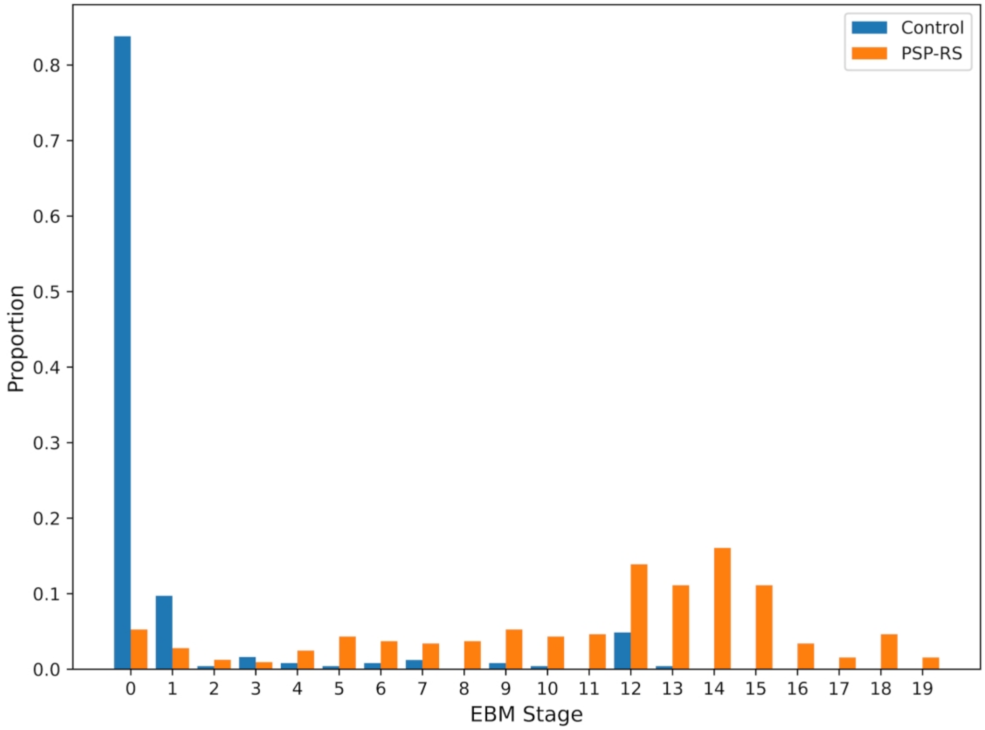

Figure 2: Histogram of event-based model staging results for PSP-RS. Healthy controls in blue and PSP-RS cases in orange. Each bar represents the proportion of patients in each category at each EBM stage. Each EBM stage on x-axis represents the occurrence of a new biomarker transition event. Stage 0 corresponds to no events having occurred and Stage 19 corresponds to all events having occurred. Events are ordered by the maximum likelihood sequence for the whole PSP-RS population as shown in Fig. 1A.

200x150mm (300 x 300 DPI)

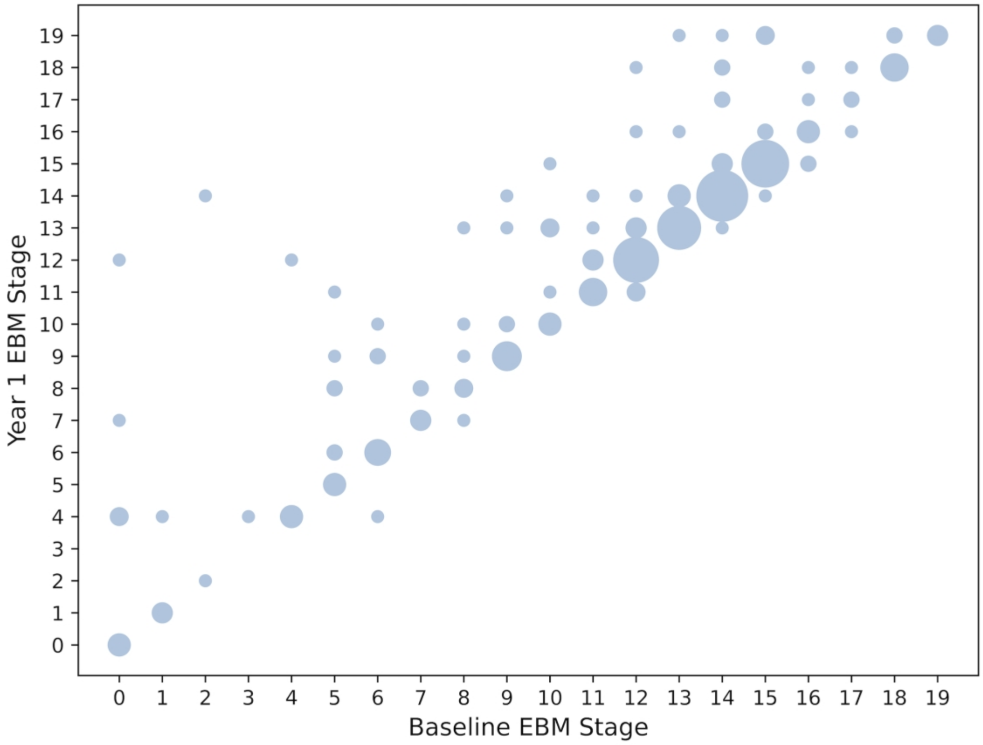

Figure 3: Longitudinal consistency of baseline EBM. Scatter plot showing predicted stage at baseline (x-axis) versus predicted stage at 12 months (y-axis) for those PSP-RS subjects with a follow-up scan (n = 255). The area of a circle is weighted by the number of subjects at each point.

175x133mm (300 x 300 DPI)

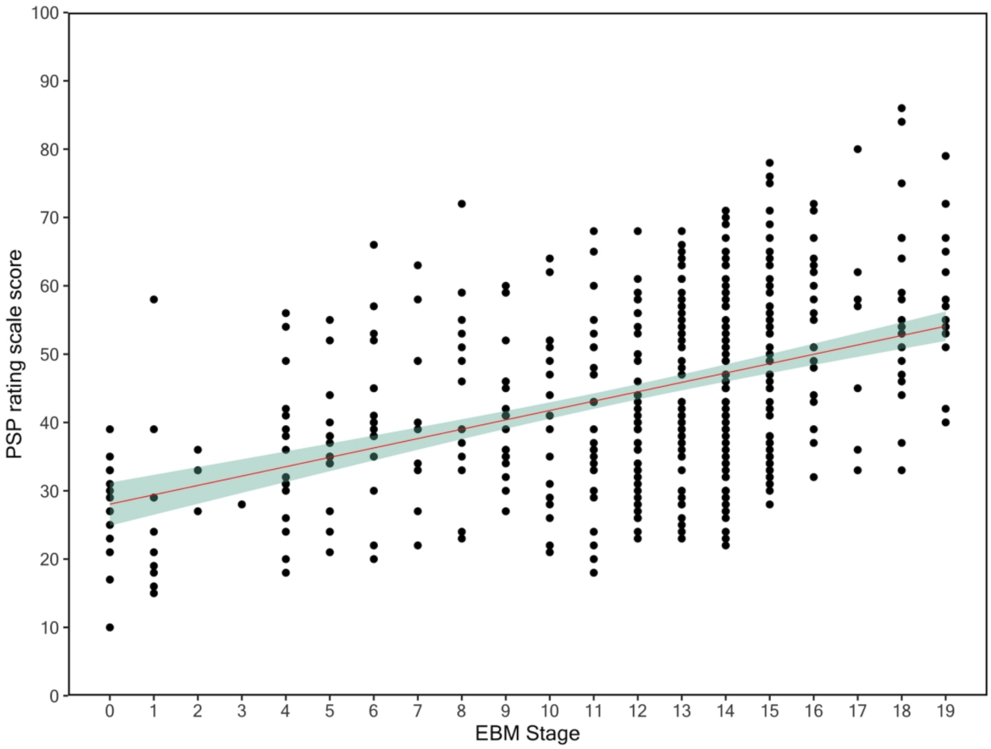

Figure 4: Association between predicted EBM stage and PSP Rating Scale score. PSP Rating Scale score versus EBM stage with line representing the linear fixed effect model fit to all subjects, and 95% confidence intervals. Subject Id was modelled as a random effect due to some subjects having two MRI scans at different time points.

178x133mm (300 x 300 DPI)

Table 1: PSP-RS EBM baseline demographics.

Baseline Demographics	PSP-RS	Controls	P value
N (12 mths)	365 (275)	289	-
Post QC - N (12 mths)	341 (255)	260	-
Gender (M/F)	176/165	112/148	0.03[a]
Age at first MRI (years [SD])	67.9 [6.8]	62.8 [9.4]	<0.001[b]
Time symptom onset to first MRI (years [SD])	4.1 [3.1]	-	-
Pathology [% PSP]	24 [92%]*	-	-
PSP Rating Scale [SD]	38.8 [12.5]**	-	-
UPDRS [SD]	30.6 [15.1]	-	-
MOCA [SD]	20.7 [5.1]	-	-

[a] Chi Square
[b] Unpaired two-tailed t-test
* % of all cases pre-QC
** 100% of cases included had a PSP rating scale score
PSP-RS = Progressive Supranuclear Palsy Richardson Syndrome

For Review Only

Details of cohorts

Supp. Table 1 summarises the basic demographics of cases from each contributing cohort.

4RTNI1 / FTLDNI trials

Participants were recruited as part of two longitudinal observational neuroimaging studies; 4RTNI which enrolled PSP and CBS patients and FTLDNI which recruited healthy controls. Both trials were managed by the University of California (UCSF) with patients also recruited at University of California of San Diego (UCSD), University of Toronto (UToronto) and Massachusetts General Hospital (MGH). A common study design and protocol was run at all sites. Patients with PSP met the NINDS-SPSP criteria (1), while CBS patients met the Amstrong criteria for possible or probable CBS-CBD subtype (2): all participants had to be aged between 45 – 90yrs to be considered for inclusion. Participants were evaluated at baseline, 6 and 12 months with an MRI scan, and a clinical assessment that included a PSPRS score, SEADL, UPDRS, MOCA and MMSE.

Three scanner types (all 3T) were used. At UCSF and MGH a Siemens Tim Trio system (Siemens, Iselin, NJ) with a 12-channel receiver head coil was used; whole brain images were acquired with a volumetric magnetization prepared rapid gradient-echo sequence (MPRAGE; repetition time (TR)/echo time (TE)/inversion time (TI) = 2300/2.98/900 ms, $\alpha = 9°$, 1 x 1 mm in-plane resolution, 1 mm slice thickness). Scans at UCSD were acquired on a GE Discovery MR750 system (GE, Milwaukee, WI) equipped with 32-channel head coil, and scans at UToronto were acquired on a GE Signa HDx system equipped with an 8-channel receiver head coil. Whole brain images at both UToronto and UCSD were acquired sagittally with a 3D inversion-recovery prepared spoiled gradient echo imaging pulse sequence (UCSD IR-SPGR; TR/TE/TI = 7.1/3.00/400 ms, $\alpha = 11°$, 1 x 1 mm in-plane resolution, 1.2 mm slice thickness;

For Review Only

UToronto IR-SPGR; TR/TE/TI = 7.0/2.80/400 ms, α = 11°, 1 x 1 mm in-plane resolution, 1.2 mm slice thickness) (3). For each patient, baseline and follow-up MRI were acquired on the same scanner using the same sequence parameters.

DAV trial

Patients were recruited from 48 centres in Australia, Canada, France, Germany, the UK, and the USA, between September 2010 and Novemeber 2012 [143]. For inclusion participants had to be aged between 41-85 at disease onset and meet modified PSP criteria from the national Neuroprotection and Natural History in Parkinson Plus Syndromes (NNIPPS) study for the most common clinical presentation PSP-RS [139]. Specifically, they had to have at least a 12-month history of postural instability or falls during the first 3 years from disease onset, supranuclear ophthalmoplegia or reduced downward saccade velocity, and prominent axial rigidity. In addition, at time of screening participants were required to have a mini-mental state examination (MMSE) score of at least 15, be able to ambulate independently (or walk 5 steps with minimal assistance), live outside a dementia care facility, have PSP symptoms for either less than 5 years, or more than 5 years with a PSPRS score or no more than 40. More detailed inclusion and exclusion criteria are included in the original study manuscript [143].

Primary endpoints were the change in PSPRS and SEADL between baseline and twelve-month visit, with secondary outcomes including Clinical Global Impression of Change (CGIC), and MRI measured ventricular volume. For additional exploratory outcomes collected please refer to original study manuscript. MRI data was collected on forty-eight 1.5T or 3T scanners with varying scanner types but consistent sequences based on standards set by the Mayo Clinic's Aging and Dementia Imaging Research laboratory (Rochester, MN, USA). All T1 images acquired were one of MPRAGE, Coronal IR-SPGR, or Sagittal IR-SPGR. [143,174]. For each

For Review Only

patient, baseline and follow-up MRI were acquired on the same scanner using the same sequence parameters.

SAL / YP trials

The Sal trial recruited from the University of California San Francisco (UCSF; San Francisco, CA) Memory and Aging Center and the Oregon Health and Science University (Portland, OR) Parkinson Center & Movement Disorder Program between June 2015 to February 2018 (4). In the YP trial patients were recruited from UCSF, and the trial ran from June 2015 to August 2017. Individuals included in both studies had PSP-RS as defined by the 2017 International Parkinson and Movement Disorder Society criteria for PSP-RS (5), were aged 50 to 85 years; had a MMSE score of 14-30, an MRI consistent with PSP, and were on stable medications at least 1 month before screening, except for approved AD and PD medications. For more detail on inclusion and exclusion criteria please refer to original study manuscript (4).

Given these were phase 1 open label trials, the primary outcome measure was safety and tolerability. In addition, PSPRS (among other clinical scales) and an MRI were collected at baseline, and six months follow-up (after drug treatment). Structural MRIs were acquired on a 3T Siemens TimTrio or a 3T Siemens Prisma-Fit scanner (Siemens Healthineers AG, Erlangen, Germany). On the TimTrio the following acquisition protocol was used; T1 mprage sequence with slice thickness 1mm, with TR of 2.3s, TE of 2.98 ms, and T1 of 900ms. The Prisma Fit acquisition protocol was identical to that on the TimTrio. For each patient, baseline and follow-up MRI were acquired on the same scanner using the same sequence parameters.

PROSPECT trial

The PROSPECT observational study recruits' patients from seven main UK study sites; University College London (UCL), Cambridge, Oxford, Newcastle, Manchester, Brighton and

For Review Only

Newport. Recruitment started in September 2015 and is ongoing. Inclusion into the study was originally defined for PSP according to the NINDS-SPSP criteria (1). At the end of baseline recruitment all cases were reclassified according to the 2017 MDS clinical PSP diagnostic criteria (5). All PSP cases met the criteria for at least “possible” PSP, and were stratified into PSP-RS, PSP cortical (PSP-CBS, PSP-SL, PSP-F) and PSP sub-cortical (PSP-P, PSP-PGF, PSP-oculomotor) (6). CBS was diagnosed according to the Armstrong criteria (2). CBS cases with CSF biomarkers consistent with AD were classed as CBS-AD, CBS-4RT if CSF was normal and CBS-indeterminate (IDT) if CSF status / autopsy diagnosis was unknown. Recruited control participants included a spouse or a friend of the case or came through the Join Dementia Research volunteer registry (6).

Study assessments including a PSPRS score (7), a modified MDS Unified Parkinson’s Disease Rating Scale (UPDRS) (8), SEADL (9), and cognitive tests including the Montreal Cognitive Assessment (MoCA) (10) and Addenbrooke’s Cognitive Examination3 (ACE-III) (11) were performed at baseline and follow-up visits (6, 12, and 24 months). Participants had volumetric weighted MRI on Siemens 3T scanners; either a Magnetom Skyra, Magnetom Prisma, or TioTim. Scan protocols were designed at the outset of the study to closely match across centres, based on the international Genetic Frontotemporal Dementia Initiative protocols (MP-RAGE, TR 2s, TE 2.93ms, Flip angle 8 degrees, 1.1mm isotropic) (12). For each patient, baseline and follow-up MRI were acquired on the same scanner using the same sequence parameters.

UCL DRC Dementia FTD Cohort

We reviewed the UCL DRC FTD cohort MRI database to identify patients with a clinical diagnosis of either PSP or CBS, and a good quality T1-weighted MRI scan. Patients were diagnosed as PSP-RS according to the NINDS-SPSP criteria (1) if diagnosis had been before 2017, or as a PSP syndrome according to the MDS Clinical PSP criteria (5). if diagnosed from

https://mc.manuscriptcentral.com/braincom

2017 onwards. CBS patients were diagnosed according to the Amstrong Criteria (2) as probable CBS-CBD. All patients included were between the age of 42 – 85 years. All patients had initially undergone a standard clinical assessment at the National Hospital for Neurology and Neurosurgery in a specialist cognitive disorders or movement disorder clinic, depending on their initial clinical presentation. Age and gender matched controls were also identified from this database and included in our control cohort.

T1-weighted MRIs were acquired between 1992 to 2014 on three different scanners: a 1.5T Signa scanner (GE Medical systems, Milwaukee, WI, TR = 12 ms, TI = 650 ms, TE = 5 ms, acquisition matrix = 256 × 256, spatial resolution = 1.5 mm), a 3T Tim Trio (Siemens, Erlangen, Germany, TR = 2200 ms, TI = 900 ms, TE = 2.9 ms, acquisition matrix = 256 × 256, spatial resolution = 1.1 mm), and a 3T Prisma scanner (Siemens, Erlangen, Germany, TR = 2,000 ms, TI = 850 ms, TE = 2.93 ms, acquisition matrix = 256 × 256, spatial resolution = 1.1 mm, acquisition plane=sagittal).

For Review Only

Supplementary Table 1: Overview of all cohorts included in study[a].

Baseline Demographics	4RTNI	DAV	SAL/YP	Prospect	UCL	Controls[b]
N (12 mths)	62 (40)	230 (220)	14 (0)	36 (12)	23 (3)	289
Gender (M/F)	28/34	119/111	6/8	23/13	16/7	127/162
Age at first MRI (years [SD])	70.5 [7.4]	67.4 [6.6]	69.4 [4.0]	67.2 [8.5]	66.1 [4.9]	62.6 [9.8]
Time onset to first MRI (years [SD])	5.5 [3.9]	15% > 5 years[c]	-	2.9 [1.9]	3.5 [1.9]	-

[a] Case numbers in this table are before quality control. PSP-RS unless otherwise stated
[b] Control cohort consists of healthy controls from FTLDNI, Prospect and UCL with no evidence of neurological disease and otherwise fit and healthy.
[c] For cases included in Davunetide trial disease, duration was only recorded as greater or less than 5 years since disease onset.

For Review Only

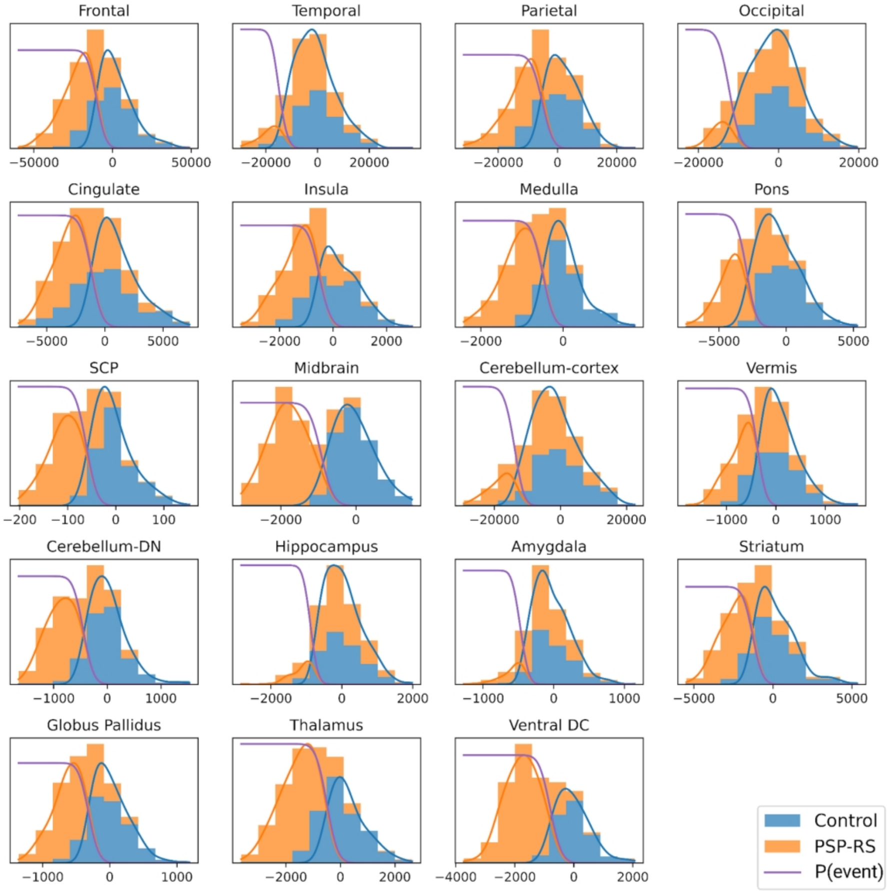

Supplementary Figure 1: Kernel Density Estimation (KDE) mixture models. Healthy controls (blue) and PSP-RS (orange) volume biomarker distributions, and corresponding KDE mixture model fits. The purple line represents the probability that an event has occurred P(x_ij |E_i). Note that the volumes are covariate corrected.

168x158mm (300 x 300 DPI)

STROBE statement: Reporting guidelines checklist for cohort, case-control and cross-sectional studies

SECTION	ITEM NUMBER	CHECKLIST ITEM	REPORTED ON PAGE NUMBER:
**TITLE AND ABSTRACT**			
	1a	Indicate the study's design with a commonly used term in the title or the abstract	1
	1b	Provide in the abstract an informative and balanced summary of what was done and what was found	1
**INTRODUCTION**			
Background and objectives	2	Explain the scientific background and rationale for the investigation being reported	4-5
	3	State specific objectives, including any pre-specified hypotheses	5
**METHODS**			
Study design	4	Present key elements of study design early in the paper	6
Setting	5	Describe the setting, locations, and relevant dates, including periods of recruitment, exposure, follow-up, and data collection	6
Participants	6a	Cohort study—Give the eligibility criteria, and the sources and methods of selection of participants. Describe methods of follow-up<br>Case-control study—Give the eligibility criteria, and the sources and methods of case ascertainment and control selection. Give the rationale for the choice of cases and controls<br>Cross-sectional study—Give the eligibility criteria, and the sources and methods of selection of participants	6 – main manuscript<br>1-5 – supplementary material
	6b	Cohort study—For matched studies, give matching criteria and number of exposed and unexposed<br>Case-control study—For matched studies, give matching criteria and the number of controls per case<br>Variables	n/a
Variables	7	Clearly define all outcomes, exposures, predictors, potential confounders, and effect modifiers. Give diagnostic criteria, if applicable	6, 7

For Review Only

SECTION	ITEM NUMBER	CHECKLIST ITEM	REPORTED ON PAGE NUMBER:
Data sources/measurements	8*	For each variable of interest, give sources of data and details of methods of assessment (measurement). Describe comparability of assessment methods if there is more than one group.	7 – main manuscript<br>1-5 – supplementary material
Bias	9	Describe any efforts to address potential sources of bias.	7
Study size	10	Explain how the study size was arrived at	6
Quantitative variables	11	Explain how quantitative variables were handled in the analyses. If applicable, describe which groupings were chosen and why .	7-10
Statistical methods	12a	Describe all statistical methods, including those used to control for confounding	7-10
	12b	Describe any methods used to examine subgroups and interactions	n/a
	12c	Explain how missing data were addressed	10
	12d	Cohort study—If applicable, explain how loss to follow-up was addressed<br>Case-control study—If applicable, explain how matching of cases and controls was addressed<br>Cross-sectional study—If applicable, describe analytical methods taking account of sampling strategy	n/a
	12e	Describe any sensitivity analyses	n/a
**RESULTS**			
Participants	13a	Report numbers of individuals at each stage of study—eg numbers potentially eligible, examined for eligibility, confirmed eligible, included in the study, completing follow-up, and analysed	Table 1<br>Supp Table 1
	13b	Give reasons for non-participation at each stage	n/a
	13c	Consider use of a flow diagram	n/a
Descriptive Data	14a	Give characteristics of study participants (eg demographic, clinical, social) and information on exposures and potential confounders	Table 1<br>Supp Table 1
	14b	Indicate number of participants with missing data for each variable of interest	11
	14c	Cohort study—Summarise follow-up time (eg, average and total amount)	n/a

For Review Only

SECTION	ITEM NUMBER	CHECKLIST ITEM	REPORTED ON PAGE NUMBER:
Outcome Data	15*	Cohort study—Report numbers of outcome events or summary measures over time<br>Case-control study—Report numbers in each exposure category, or summary measures of exposure<br>Cross-sectional study—Report numbers of outcome events or summary measures	11-13
Main Results	16a	Give unadjusted estimates and, if applicable, confounder-adjusted estimates and their precision (e.g. 95% confidence interval). Make clear which confounders were adjusted for and why they were included	7<br>13
	16b	Report category boundaries when continuous variables were categorized	n/a
	16c	If relevant, consider translating estimates of relative risk into absolute risk for a meaningful time period	n/a
	16d	Report results of any adjustments for multiple comparisons	7
Other Analyses	17a	Report other analyses done—e.g. analyses of subgroups and interactions, and sensitivity analyses	13
	17b	If numerous genetic exposures (genetic variants) were examined, summarize results from all analyses undertaken	n/a
	17c	If detailed results are available elsewhere, state how they can be accessed	n/a
**DISCUSSION**			
Key Results	18	Summarise key results with reference to study objectives	13-14
Limitations	19	Discuss limitations of the study, taking into account sources of potential bias or imprecision. Discuss both direction and magnitude of any potential bias	16-17
Interpretation	20	Give a cautious overall interpretation of results considering objectives, limitations, multiplicity of analyses, results from similar studies, and other relevant evidence	18
Generalisability	21	Discuss the generalisability (external validity) of the study results<br>Other information	17-18
**FUNDING**			
	22	Give the source of funding and the role of the funders for the present study and, if applicable, for the original study on which the present article is based	18-20

For Review Only

*Give information separately for cases and controls in case-control studies and, if applicable, for exposed and unexposed groups in cohort and cross-sectional studies.

For Review Only
