# Supplementary material for: A data-driven model of brain volume changes in progressive supranuclear palsy
Source: Brain Commun. 2022 Apr 14;4(3):fcac098. doi: 10.1093/braincomms/fcac098 (PMC9118104; doi:10.1093/braincomms/fcac098)
Supplement: fcac098_Supplementary_Data [file fcac098_supplementary_data.zip › Supplementary_Figure_Legends.docx]

**Supplementary Figure 1**: **Kernel Density Estimation (KDE) mixture models.** Healthy controls (blue) and PSP-RS (orange) volume biomarker distributions, and corresponding KDE mixture model fits. The purple line represents the probability that an event has occurred $P\left( x_{ij}|E_{i} \right)$. Note that the volumes are covariate corrected.

**Supplementary Figure 2: Sequence of PSP-RS atrophy progression after 5-fold cross validation.** Re-estimation of positional variance after cross-validation of the maximum likelihood event sequence across 50-folds (10 repeats and 5-folds). The vertical ordering on the y-axis (from top to bottom) shows the maximum likelihood sequence estimated by the EBM (earliest to latest event). The bottom x-axis shows EBM stage while the top x-axis represents the percentage of regions atrophic (abnormal) at each stage. Colour intensity of the squares represents the posterior confidence in each biomarker’s position in the sequence after cross-validation. SCP = superior cerebellar peduncle, Ventral DC = ventral diencephalon. Note that because these volumes are covariate adjusted the control distribution will be centred at zero.

**Supplementary Figure 3:**  **Association between predicted EBM stage, PSPRS and disease duration at baseline scan. (A)** PSP Rating Scale score versus EBM stage* (β=1.14, 95% CI 0.84-1.44, p<0.001, adjusted R2 0.18). **(B)** Disease duration (years) vs EBM stage** (β=0.25, 95% CI 0.20-0.30, p<0.001, adjusted R2 0.39). For both **(A)** and **(B)** the line represents the linear model fit with 95% confidence intervals.

* 241 baseline scans with PSPRS score

** 87 baseline scans with disease duration

**Supplementary Figure 4:** Linear models to test association between age at scan and predicted EBM stage. **(A)** for cases (β=0.19, 95% CI=0.13-0.25, p=0.12, adjusted R^2^=0.017) **(B)** for controls (β=-0.27, 95% CI=-0.66-0.12, p=0.18, adjusted R^2^=0.003). For **(A)** and **(B)** the line represents the linear model fit with 95% confidence intervals.
