# Supplementary material for: A data-driven model of brain volume changes in progressive supranuclear palsy
Source: Brain Commun. 2022 Apr 14;4(3):fcac098. doi: 10.1093/braincomms/fcac098 (PMC9118104; doi:10.1093/braincomms/fcac098)
Supplement: fcac098_Supplementary_Data [file fcac098_supplementary_data.zip › Supplementary_Material.docx]

# Details of cohorts

### 4RTNI1 / FTLDNI trials

Participants were recruited as part of two longitudinal observational neuroimaging studies; 4RTNI which enrolled PSP and CBS patients and FTLDNI which recruited healthy controls. Both trials were managed by the University of California (UCSF) with patients also recruited at University of California of San Diego (UCSD), University of Toronto (UToronto) and ﻿Massachusetts General Hospital (MGH). A common study design and protocol was run at all sites. Patients with PSP met the NINDS-SPSP criteria (1), while CBS patients met the Amstrong criteria for possible or probable CBS-CBD subtype (2): all participants had to be aged between 45 – 90yrs to be considered for inclusion. Participants were evaluated at baseline, 6 and 12 months with an MRI scan, and a clinical assessment that included a PSPRS score, SEADL, UPDRS, MOCA and MMSE.

Three scanner types (all 3T) were used. At UCSF and MGH a Siemens Tim Trio system (Siemens, Iselin, NJ) with a 12-channel receiver head coil was used; whole brain images were acquired with a volumetric magnetization prepared rapid gradient-echo sequence (MPRAGE; repetition time (TR)/echo time (TE)/inversion time (TI) = 2300/2.98/900 ms, α = 9°, 1 x 1 mm in-plane resolution, 1 mm slice thickness). Scans at UCSD were acquired on a GE Discovery MR750 system (GE, Milwaukee, WI) equipped with 32-channel head coil, and scans at UToronto were acquired on a GE Signa HDx system equipped with an 8-channel receiver head coil. Whole brain images at both UToronto and UCSD were acquired sagittally with a 3D inversion-recovery prepared spoiled gradient echo imaging pulse sequence (UCSD IR-SPGR; TR/TE/TI = 7.1/3.00/400 ms, α = 11°, 1 x 1 mm in-plane resolution, 1.2 mm slice thickness; UToronto IR-SPGR; TR/TE/TI = 7.0/2.80/400 ms, α = 11°, 1 x 1 mm in-plane resolution, 1.2 mm slice thickness) (3). For each patient, baseline and follow-up MRI were acquired on the same scanner using the same sequence parameters.

### DAV trial

Patients were recruited from 48 centres in Australia, Canada, France, Germany, the UK, and the USA, between September 2010 and Novemeber 2012 ^143^. For inclusion participants had to be aged between 41-85 at disease onset and meet modified PSP criteria from the national Neuroprotection and Natural History in Parkinson Plus Syndromes (NNIPPS) study for the most common clinical presentation PSP-RS ^139^. Specifically, they had to have at least a 12-month history of postural instability or falls during the first 3 years from disease onset, supranuclear ophthalmoplegia or reduced downward saccade velocity, and prominent axial rigidity. In addition, at time of screening participants were required to have a ﻿mini-mental state examination (MMSE) score of at least 15, be able to ambulate independently (or walk 5 steps with minimal assistance), live outside a dementia care facility, have PSP symptoms for either less than 5 years, or more than 5 years with a PSPRS score or no more than 40. More detailed inclusion and exclusion criteria are included in the original study manuscript ^143^.

Primary endpoints were the change in PSPRS and SEADL between baseline and twelve-month visit, with secondary outcomes including ﻿Clinical Global Impression of Change (CGIC), and MRI measured ventricular volume. For additional exploratory outcomes collected please refer to original study manuscript. MRI data was collected on forty-eight 1.5T or 3T scanners with varying scanner types but consistent sequences based on standards set by the Mayo Clinic’s Aging and Dementia Imaging Research laboratory (Rochester, MN, USA). All T1 images acquired were one of MPRAGE, Coronal IR-SPGR, or Sagittal IR-SPGR. ^143,174^. For each patient, baseline and follow-up MRI were acquired on the same scanner using the same sequence parameters.

### SAL / YP trials

The Sal trial recruited from the University of California San Francisco (UCSF; San Francisco, CA) Memory and Aging Center and the Oregon Health and Science University (Portland, OR) Parkinson Center & Movement Disorder Program between June 2015 to February 2018 (4). ﻿In the YP trial patients were recruited from UCSF, and the trial ran from June 2015 to August 2017. Individuals included in both studies had PSP-RS as defined by the 2017 International Parkinson and Movement Disorder Society criteria for PSP-RS (5), were aged 50 to 85 years; had a MMSE score of 14-30, an MRI consistent with PSP, and were on stable medications at least 1 month before screening, except for approved AD and PD medications. For more detail on inclusion and exclusion criteria please refer to original study manuscript (4).

Given these were phase 1 open label trials, the primary outcome measure was safety and tolerability. In addition, PSPRS (among other clinical scales) and an MRI were collected at baseline, and six months follow-up (after drug treatment). ﻿Structural MRIs were acquired on a 3T Siemens TimTrio or a 3T Siemens Prisma-Fit scanner (Siemens Healthineers AG, Erlangen, Germany). On the TimTrio the following acquisition protocol was used; T1 mprage sequence with slice thickness 1mm, with TR of 2.3s, TE of 2.98 ms, and T1 of 900ms. The Prisma Fit acquisition protocol was identical to that on the TimTrio. For each patient, baseline and follow-up MRI were acquired on the same scanner using the same sequence parameters.

### PROSPECT trial

The PROSPECT observational study recruits’ patients from seven main UK study sites; University College London (UCL), Cambridge, Oxford, Newcastle, Manchester, Brighton and Newport. Recruitment started in September 2015 and is ongoing. Inclusion into the study was originally defined for PSP according to the NINDS-SPSP criteria (1). At the end of baseline recruitment all cases were reclassified according to the 2017 MDS clinical PSP diagnostic criteria (5). All PSP cases met the criteria for at least “possible” PSP, and were stratified into PSP-RS, PSP cortical (PSP-CBS, PSP-SL, PSP-F) and PSP sub-cortical (PSP-P, PSP-PGF, PSP-oculomotor) (6). CBS was diagnosed according to the Armstrong criteria (2). CBS cases with CSF biomarkers consistent with AD were classed as CBS-AD, CBS-4RT if CSF was normal and CBS-indeterminate (IDT) if CSF status / autopsy diagnosis was unknown. Recruited control participants included a spouse or a friend of the case or came through the Join Dementia Research volunteer registry (6).

Study assessments including a PSPRS score (7), a modified MDS Unified Parkinson’s Disease Rating Scale (UPDRS) (8), SEADL (9), and cognitive tests including the Montreal Cognitive Assessment (MoCA) (10) and Addenbrooke’s Cognitive Examination3 (ACE-III) (11) were performed at baseline and follow-up visits (6, 12, and 24 months). Participants had volumetric weighted MRI on Siemens 3T scanners; either a Magnetom Skyra, Magnetom Prisma, or TioTim. Scan protocols were designed at the outset of the study to closely match across centres, based on the international Genetic Frontotemporal Dementia Initiative protocols (MP-RAGE, TR 2s, TE 2.93ms, Flip angle 8 degrees, 1.1mm isotropic) (12). For each patient, baseline and follow-up MRI were acquired on the same scanner using the same sequence parameters.

### UCL DRC Dementia FTD Cohort

We reviewed the UCL DRC FTD cohort MRI database to identify patients with a clinical diagnosis of either PSP or CBS, and a good quality T1-weighted MRI scan. Patients were diagnosed as PSP-RS according to the NINDS-SPSP criteria (1) if diagnosis had been before 2017, or as a PSP syndrome according to the MDS Clinical PSP criteria (5). if diagnosed from 2017 onwards. CBS patients were diagnosed according to the Amstrong Criteria (2) as probable CBS-CBD. All patients included were between the age of 42 – 85 years. All patients had initially undergone a standard clinical assessment at the National Hospital for Neurology and Neurosurgery in a specialist cognitive disorders or movement disorder clinic, depending on their initial clinical presentation. Age and gender matched controls were also identified from this database and included in our control cohort.

T1-weighted MRIs were acquired between 1992 to 2014 on three different scanners: a 1.5T Signa scanner (﻿GE Medical systems, Milwaukee, WI, TR = 12 ms, TI = 650 ms, TE = 5 ms, acquisition matrix = 256 × 256, spatial resolution = 1.5 mm), a 3T Tim Trio (﻿Siemens, Erlangen, Germany, TR = 2200 ms, TI = 900 ms, TE = 2.9 ms, acquisition matrix = 256 × 256, spatial resolution = 1.1 mm), and a 3T Prisma scanner (Siemens, Erlangen, Germany, TR = 2,000 ms, TI = 850 ms, TE = 2.93 ms, acquisition matrix = 256 × 256, spatial resolution = 1.1 mm, acquisition plane=sagittal).

**TABLES**

**Supp. Table 1** summarises the basic demographics of cases from each contributing cohort.

﻿
