# Supplementary material for: A data-driven model of brain volume changes in progressive supranuclear palsy
Source: Brain Commun. 2022 Apr 14;4(3):fcac098. doi: 10.1093/braincomms/fcac098 (PMC9118104; doi:10.1093/braincomms/fcac098)
Supplement: fcac098_Supplementary_Data [file fcac098_supplementary_data.zip › SupplementaryTable1.docx]

**Supplementary Table 1: Overview of all cohorts included in study^a^.**

| **Baseline Demographics** | **4RTNI** | **DAV** | **SAL/YP** | **Prospect** | **UCL** | **Controls^b^** |
| --- | --- | --- | --- | --- | --- | --- |
| N (12 mths) | 62 (40) | 230 (220) | 14 (0) | 36 (12) | 23 (3) | 289 |
| Gender (M/F) | 28/34 | 119/111 | 6/8 | 23/13 | 16/7 | 127/162 |
| Age at first MRI (years [SD]) | 70.5 [7.4] | 67.4 [6.6] | 69.4 [4.0] | 67.2 [8.5] | 66.1 [4.9] | 62.6 [9.8] |
| Time onset to first MRI (years [SD]) | 5.5 [3.9] | 15% > 5 years^c^ | - | 2.9 [1.9] | 3.5 [1.9] | - |

^a^ Case numbers in this table are before quality control. PSP-RS unless otherwise stated

^b^ Control cohort consists of healthy controls from FTLDNI, Prospect and UCL with no evidence of neurological disease and otherwise fit and healthy.

^c^ For cases included in Davunetide trial disease, duration was only recorded as greater or less than 5 years since disease onset.
