# Supplementary material for: A data-driven model of brain volume changes in progressive supranuclear palsy
Source: Brain Commun. 2022 Apr 14;4(3):fcac098. doi: 10.1093/braincomms/fcac098 (PMC9118104; doi:10.1093/braincomms/fcac098)
Supplement: fcac098_Supplementary_Data [file Original Submission - Decision Letter.pdf › Original Submission - Decision Letter.pdf]

Original Submission – Decision Letter

11-Oct-2021

Dear Dr. Scotton,

Thank you for your recent submission BRAINCOM-2021-322
entitled "A data-driven model of brain volume changes in Progressive Supranuclear Palsy" to Brain Communications.

Your manuscript has been reviewed, and while the reviewers had largely positive comments, they have some constructive critiques that I think you could address in a minor revision of the manuscript. Therefore, I invite you to respond to the reviewers' comments and revise your manuscript. The reviewers' comments are included at the bottom of this message.

In addition to the reviewers' comments, please also address our Scientific Editor's comments added below to comply with our requirements for presentation of scientific data at Brain Communications.

Please format your revised manuscript according to our Instructions to Authors https://academic.oup.com/braincomms/pages/General_Instructions – a summary of key points is attached to help with this.

To revise your manuscript, click the link below or log into https://mc.manuscriptcentral.com/braincom and enter your Author Centre, where you will find your manuscript title listed under "Manuscripts with Decisions." Under "Actions," click on "Create a Revision." Your manuscript number will be appended to denote a revision.

*** PLEASE NOTE: This is a two-step process. After clicking on the link, you will be directed to a webpage to confirm. ***

https://mc.manuscriptcentral.com/braincom?URL_MASK=90362abf84c14609a89fe5d07794af89

Please upload a revised manuscript with the changes you have made shown either in coloured text or using "track changes".

When submitting your revised manuscript, you will be asked to respond to the reviewer(s) comments. Please be as specific as possible in your response to all of the comments.

The editorial team at Brain Communications also aims to increase transparency in scientific publishing and to demonstrate the value that peer review brings to the scientific process. Please therefore consider whether you are willing to have reviewer comments on your paper and your response published as supplementary data with the paper, if it is accepted. This is entirely optional and will only be done if both the authors and reviewers agree. When submitting your revision you will have the opportunity to update the answer you entered previously.

IMPORTANT: Your original files are available to you when you upload your revised manuscript. Please delete any redundant files before completing the submission.

Because we are trying to facilitate timely publication of manuscripts submitted to Brain Communications, we would appreciate your revised manuscript as soon as possible. If it is not possible for you to resubmit within 3 months, the option to submit a revision will expire, and we would then consider your revised paper as a new submission. However, if you need a little more time, please get in touch and we can extend this deadline for you.

Once again, thank you for submitting your manuscript to Brain Communications and I look forward to receiving your revision.

Best wishes

Dr. Graciela Muniz Terrera
Associate Editor, Brain Communications

Professor Tara Spires-Jones
Founding Editor, Brain Communications

Reviewer(s)' Comments to Author:

Reviewer: 1 (Günter Höglinger)

Comments to the Author
The authors assembled a large cohort of PSP-RS patients with crossectional and longitudinal MRI datasets available. Using a data-driven approach, they developed a staging system for sequentially allocating patients into disease stages in sequential order, defined by thresholded atrophy in disease-associated brain regions. The methodological approach and clarity of presentation are impressive.

Some aspects might further improve the work:

- One relevant limitation is the significant difference in age between cases and controls (68 vs 63 yrs). Due to atrophy during normal ageing, this difference is very relevant and may have affected the atrophy thresholds defining 'events'. How would data change if cases and controls were matched by age?

- Page 13, line 20: Authors suggest that cases with decreased volumes in regions including the hippocampus with relative sparing of the brainstem and subcortical structures may represent people with preclinical Alzheimer’s disease. are there biomarker data to support this assumption?

- Fig. 1: when reaching stage x [x=1-19], defined by event x (= atrophy in region x), what percentage of regions y (y= 1- (x-1)) were indeed atrophic. Please display % as function of x.

- Fig 4: please add panels:
  - EBM stage in cases as function of disease duration (where available)
  - EBM stage in cases as function of age
  - EBM stage in CONTROLS as function of age

Reviewer: 2 (Minming Zhang)

Comments to the Author
In this study, William et al explored the progression pattern of PSP by using a very popular data-driven model (EBM). They used the EBM to estimate a sequence of brain atrophy in PSP and form an objective in vivo staging system which mirrors the recent post-mortem brain pathology staging system. The MRI-based fine-grained staging system also correlates with clinical measures of disease severity. In addition, the longitudinal validation was conducted.
This is a well-designed study with clear research objectives and large samples size. The correlation between EBM stage and disease severity, and the high classification accuracy of EBM stage are interesting and important, which indicate that the structural MRI is suitable for reflecting the pathology of PSP-RS. However, some sections could benefit from clarification. I have the following minor comments:

1. Methods—Biomarker selection: Is there some basis for setting the threshold to Bonferroni corrected $p < 0.0001$? To keep the effect size? Or suitable for KDE model?

2. Methods—The selection of healthy controls: Based on practical experience, A variety of atrophy could also show in healthy controls. Are there some specific screenings for healthy controls in your study? Whether it is necessary to exclude the healthy control with severe atrophy before conducting EBM analysis?

3. Methods—Cross-validation: We noted that the author used the 100 bootstrap samples of the original data for cross validation. This method is usually used in small sample size. Why not use 5-fold or 10-fold cross-validation with this large sample size?

4. Results: Is baseline EBM stage correlated with the PSP rating score at baseline? This is also very important.

5. Results: It is known that neurodegenerative diseases have great heterogeneity. Is there are different subtypes in PSP-RS? The EBM was established on the probability of biomarkers. Thus, a great heterogeneity (different progress patterns) can cause fault progress sequences inference. We wonder that the SuStaIn subtype method (Young AL et al, 2018) could be useful in solving this problem. Maybe you can test whether there are different progress pattern using this method. Or explain this.

6. Results: We also want to know the diagnosis accuracy of PSP-RS using the ROI biomarkers directly. Is it higher or lower than EBM stage?

7. Introduction: Third paragraph '…with additional involvement of the subcortical structures.' Should change 'subcortical structures' to 'cortical structures'.

Comments from the Scientific Editor:

-Please supply a graphical abstract. A graphical abstract is a visual representation of the central finding or methodology of the paper. It will be displayed in the table of contents. Authors should strive to make the graphical abstract informative, interesting, visually appealing, and straightforward (no caption needed, no abbreviations except gene names). Please upload as a separate high resolution image file (tiff preferred). The technical requirements for the graphical abstracts are:
--size of the submitted image: 1200 pixels square at 300dpi
--font of the text: Arial 12-16 point (smaller fonts will not be legible online)

-Please add an Abbreviated Summary (up to 50 words) of your paper that captures the main purpose and conclusions of the work. The summary should state the main results written in the third person ('[1st author surname] et al. report that …') and conclusion. This summary will be used in the contents list online and should not contain any abbreviations other than those on the accepted abbreviations list and accepted gene/protein names, making it accessible to the non-specialist. Please upload it as a Word document. Allowed abbreviations: AIDS; ANOVA; AMPA; ATP; A,T,C,G; CNS; CSF; CT; DNA; ECG; EEG; EMG; GABA; HIV; MRI; NMDA; PET; PCR; RNA.

-please report in the figure legend the test used and relevant test statistic values alongside p values (for example F or t values for ANOVAs and t-tests).
