# Supplementary material for: A data-driven model of brain volume changes in progressive supranuclear palsy
Source: Brain Commun. 2022 Apr 14;4(3):fcac098. doi: 10.1093/braincomms/fcac098 (PMC9118104; doi:10.1093/braincomms/fcac098)
Supplement: fcac098_Supplementary_Data [file Original Submission -Author's Response.pdf › Original Submission -Author's Response.pdf]

11-Oct-2021

Dear Dr. Scotton,

Thank you for your recent submission BRAINCOM-2021-322
entitled "A data-driven model of brain volume changes in Progressive Supranuclear Palsy" to Brain Communications.

Your manuscript has been reviewed, and while the reviewers had largely positive comments, they have some constructive critiques that I think you could address in a minor revision of the manuscript. Therefore, I invite you to respond to the reviewers' comments and revise your manuscript. The reviewers' comments are included at the bottom of this message.

In addition to the reviewers' comments, please also address our Scientific Editor's comments added below to comply with our requirements for presentation of scientific data at Brain Communications.

Please format your revised manuscript according to our Instructions to Authors https://academic.oup.com/braincomms/pages/General_Instructions – a summary of key points is attached to help with this.

To revise your manuscript, click the link below or log into https://mc.manuscriptcentral.com/braincom and enter your Author Centre, where you will find your manuscript title listed under "Manuscripts with Decisions." Under "Actions," click on "Create a Revision." Your manuscript number will be appended to denote a revision.

*** PLEASE NOTE: This is a two-step process. After clicking on the link, you will be directed to a webpage to confirm. ***

https://mc.manuscriptcentral.com/braincom?URL_MASK=90362abf84c14609a89fe5d07794af89

Please upload a revised manuscript with the changes you have made shown either in coloured text or using "track changes".

When submitting your revised manuscript, you will be asked to respond to the reviewer(s) comments. Please be as specific as possible in your response to all of the comments.

The editorial team at Brain Communications also aims to increase transparency in scientific publishing and to demonstrate the value that peer review brings to the scientific process. Please therefore consider whether you are willing to have reviewer comments on your paper and your response published as supplementary data with the paper, if it is accepted. This is entirely optional and will only be done if both the authors and reviewers agree. When submitting your revision you will have the opportunity to update the answer you entered previously.

IMPORTANT: Your original files are available to you when you upload your revised manuscript. Please delete any redundant files before completing the submission.

Because we are trying to facilitate timely publication of manuscripts submitted to Brain Communications, we would appreciate your revised manuscript as soon as possible.   If it is not possible for you to resubmit within 3 months, the option to submit a revision will expire, and we would then consider your revised paper as a new submission. However, if you need a little more time, please get in touch and we can extend this deadline for you.

Once again, thank you for submitting your manuscript to Brain Communications and I look forward to receiving your revision.

Best wishes

Dr. Graciela Muniz Terrera
Associate Editor, Brain Communications
g.muniz@ed.ac.uk

Professor Tara Spires-Jones
Founding Editor, Brain Communications
Tara.Spires-Jones@ed.ac.uk

Dear Dr Muniz Terrera and Professor Spires-Jones,

We would like to thank the Editors and Reviewers for their comments, which were insightful and helped us to improve our manuscript. We have endeavoured to address all the reviewers' and editor's comments in our revision and response letter with references to updated text and additional figures in the manuscript and supplementary materials where appropriate.

All the authors have read and approved this version of the article, and neither the entire paper nor any part of its content has been published, accepted, or submitted to any other journal.

Yours Faithfully,

Dr William Scotton

Reviewer(s)' Comments to Author:

Reviewer: 1

Comments to the Author
The authors assembled a large cohort of PSP-RS patients with crossectional and longitudinal MRI datasets available. Using a data-driven approach, they developed a staging system for sequentially allocating patients into disease stages in sequential order, defined by thresholded atrophy in disease-associated brain regions. The methodological approach and clarity of presentation are impressive.

We would like to thank you for your positive comments regarding this work, and the opportunity to improve the manuscript. We have responded to each of your suggestions below with reference to the updated text and figures where appropriate

Some aspects might further improve the work:

- One relevant limitation is the significant difference in age between cases and controls (68 vs 63 yrs). Due to atrophy during normal ageing, this difference is very relevant and may have affected the atrophy thresholds defining 'events'. How would data change if cases and controls were matched by age?
We acknowledge the difference in average age between the controls and patient group. To account for this, we included age as a covariate in a linear regression on the control cohort, to adjust volumes of cases and controls accordingly (Page 7.). As per your comment below (*"Fig 4: please add panels.."*) we have performed additional analyses to demonstrate that age has been effectively regressed out of the volumes of both cases and controls; there is no association between EBM stage and age for either group. The Methods, Results and Discussion section have been updated accordingly.

- **METHODS:**
  - **(Pg 7):** "Linear regressions of age against predicted EBM stage were also performed (after EBM model fitting) for cases and controls separately to confirm that there was no residual correlation after adjustment."
- **RESULTS:**
  - **(Pg 14):** *"To check that we had adequately adjusted for age we also ran linear models of age as a function of predicted EBM stage for cases (Supp. Fig. 4A) and controls separately (Supp. Fig. 4B). There was no association between EBM stage and age in either the case (β=0.19, 95% CI=0.13-0.25, p=0.12, adjusted R2=0.017) or control group (β=-0.27, 95% CI=-0.66-0.12, p=0.18, adjusted R2=0.003)."*
- **DISCUSSION:**
  - **(Pg 17): "***Linear modelling of age against predicted EBM stage for cases and controls (Supp. Fig. 4 A/B) showed no association supporting the validity of this approach."*

- Page 13, line 20: Authors suggest that cases with decreased volumes in regions including the hippocampus with relative sparing of the brainstem and subcortical structures may

represent people with preclinical Alzheimer's disease. are there biomarker data to support this assumption?
It would be interesting to investigate this further. Unfortunately, we do not have access to plasma samples for say pT81 or pT217, and amyloid PET was not undertaken. This means that we are unable to test the hypothesis. We feel it is appropriate to include these participants as normal ageing, and in keeping with the study protocol for which the criteria for inclusion included "*no known diagnosis of a neurological or neurodegenerative condition, and no known history of memory complaints."* **We comment on this on page 6 to make this clear ("*Controls were defined as no known diagnosis of a neurological or neurodegenerative condition, and no known history of memory complaints."*)**

- Fig. 1: when reaching stage x [x=1-19], defined by event x (= atrophy in region x), what percentage of regions y (y= 1- (x-1)) were indeed atrophic. Please display % as function of x.
Thank-you for this suggestion. We have added an additional x-axis on the top of all positional variance diagram figures (Fig. 1A / B, Supp. Fig. 2) to indicate this information.

- Fig 4: please add panels:
  - EBM stage in cases as function of disease duration (where available) **(Added to Fig. 4 as Fig 4.B)**
  - EBM stage in cases as function of age **(Added to new figure, Supp. Fig. 4A)**
  - EBM stage in CONTROLS as function of age **(Added to new figure, Supp. Fig. 4B)**

Thank-you for this suggestion we agree this is useful information to add to the manuscript. We have carried out these analyses and produced the relevant figures. To be consistent with Figure 4 we have modelled disease duration as a function of predicted EBM stage using the same linear mixed model approach that was used in figure 4 for PSPRS score. Fig. 4 has been updated with Fig. 4a showing PSPRS and Fig. 4b. showing disease duration. The Methods, Results and Discussion section updated accordingly.

- **ABSTRACT:**
  - **(Pg 1): "***In addition, both clinical severity (PSP Rating Scale) and disease duration were significantly correlated with predicted subject EBM stage (p<0.01)."*
- **METHODS:**
  - **(Pg 10):** *"with **both** increasing PSP Rating Scale score **(the main clinical measure of disease severity) and also disease duration"***
  - **(Pg 10):** *" In addition, we analysed disease duration (time from first symptom to MRI scan) as a function of predicted EBM stage (87 baseline and 43 12-month follow-up scans had disease duration recorded) using the same method. To confirm that baseline EBM stage was also correlated with both PSPRS score and disease duration we fitted a linear model for each as a function of EBM stage."*
- **RESULTS:**
  - **(Pg 14):** *"We then modelled disease duration (years) as a function of predicted EBM stage, which showed a significant fixed effect (ß=0.29, 95% CI 0.24-0.34, p<0.001) and a conditional R2 of 0.68 (Fig. 4B). When fitting linear models for PSPRS score and disease duration versus predicted EBM stage on baseline scans only (Supp. Fig 3A / B respectively), there was also a significant*

association albeit with a lower adjusted R2 (PSPRS vs EBM stage at baseline: β=1.14, 95% CI 0.84-1.44, p<0.001, adjusted R2 0.18, disease duration vs EBM stage at baseline: (β=0.25, 95% CI 0.20-0.30, p<0.001, adjusted R2 0.39)."

- **DISCUSSION:**
  - **(Pg 14) "***The model derived staging correlated with* ***both*** *clinical severity* ***and disease duration."***
  - **(Pg 16)** *"This is supported by the association between EBM stage and disease duration in those subjects for which disease duration was recorded (Fig. 4B)"*
  - **(Pg 19): "***It provides an objective, in-vivo staging system that is longitudinally consistent and correlates with clinical measures of disease severity* ***and disease duration."***

We have also modelled age for cases and controls separately using a linear model which showed no association between age and EBM stage for either group. These figures are included in the Supp. Fig 4 (A and B respectively), and Methods, Results and Discussion section updated accordingly.

- **METHODS:**
  - **(Pg 7):** *"Linear regressions of age against predicted EBM stage were also performed (after EBM model fitting) for cases and controls separately to confirm that there was no residual correlation after adjustment."*
- **RESULTS:**
  - **(Pg 14):** *"To check that we had adequately adjusted for age we also ran linear models of age as a function of predicted EBM stage for cases (Supp. Fig. 4A) and controls separately (Supp. Fig. 4B). There was no association between EBM stage and age in either the case (β=0.19, 95% CI=0.13-0.25, p=0.12, adjusted R2=0.017) or control group (β=-0.27, 95% CI=-0.66-0.12, p=0.18, adjusted R2=0.003)."*
- **DISCUSSION:**
  - **(Pg 17): "***Linear modelling of age against predicted EBM stage for cases and controls (Supp. Fig. 4 A/B) showed no association supporting the validity of this approach."*

Reviewer: 2

Comments to the Author
In this study, William et al explored the progression pattern of PSP by using a very popular data-driven model (EBM). They used the EBM to estimate a sequence of brain atrophy in PSP and form an objective in vivo staging system which mirrors the recent post-mortem brain pathology staging system. The MRI-based fine-grained staging system also correlates with clinical measures of disease severity. In addition, the longitudinal validation was conducted.
This is a well-designed study with clear research objectives and large samples size. The correlation between EBM stage and disease severity, and the high classification accuracy of EBM stage are interesting and important, which indicate that the structural MRI is suitable

for reflecting the pathology of PSP-RS. However, some sections could benefit from clarification. I have the following minor comments:

We would like to thank the reviewer for the helpful comments to improve this work. We have addressed each of your comments below and updated the manuscript where appropriate.

1. Methods—Biomarker selection: Is there some basis for setting the threshold to Bonferroni corrected p < 0.0001? To keep the effect size? Or suitable for KDE model?

Apologies if this part was not clear. The regions of interest were selected to match most closely those used by Kovacs in their 2020 pathology staging system. We performed a two-tail unpaired t-test to check whether there was a significant difference between cases and controls for each ROI and given there were 19 ROIs we used a Bonferroni corrected threshold (0.05 / 19 = 0.003). We have updated the text on page 8 with "$p < 3 \times 10^{-3}$" to represent that all ROI effect sizes were less than the Bonferroni threshold. Thank-you for picking this up. All the pre-selected ROIs passed the corrected significance threshold. Regarding the KDE model it does not matter, *per se*, whether there is a significant effect for a ROI, but where no significant difference exists in an ROI, this ROI is not typically informative of the EBM sequence.

2. Methods—The selection of healthy controls: Based on practical experience, A variety of atrophy could also show in healthy controls. Are there some specific screenings for healthy controls in your study? Whether it is necessary to exclude the healthy control with severe atrophy before conducting EBM analysis?

Thank-you for this interesting comment. In the absence of autopsy confirmed controls (no neurodegenerative pathology at post-mortem), controls were defined as no known diagnosis of a neurological or neurodegenerative condition, and no known history of memory complaints. We have added this text on pg. 6 to make this clear ("*Controls were defined as no known diagnosis of a neurological or neurodegenerative condition, and no known history of memory complaints.").* **Although we appreciate that a small number may have an early pre-symptomatic neurodegenerative process (this is most likely to be Alzheimer's disease given the prevalence of 5.3% in people aged the 65 – 74** https://doi.org/10.1002/alz.12328**), in the absence of AD biomarker data in the control cohort, we were unable to screen out these cases before analysis. Given that most clinical trials use the same definition of healthy controls as we used in this study, it is our preferred option to base control selection on the criteria stated, rather than a "super-healthy" subgroup that are screened for pre-symptomatic pathology.**

3. Methods—Cross-validation: We noted that the author used the 100 bootstrap samples of the original data for cross validation. This method is usually used in small sample size. Why not use 5-fold or 10-fold cross-validation with this large sample size?
We agree that this is also an appropriate approach to assess model validity. In response e have performed 5 fold cross validation as detailed previously by Oxtoby et al. (Brain . 2021 Apr 12;144(3):975-988. doi: 10.1093/brain/awaa461.), to generate a positional variance diagram averaged across the 5 folds. This is included in the Supplementary

materials as Supp. Fig. 2 and shows the overall sequence of events is maintained with similar uncertainty in position to the bootstrapping method (Fig. 1b). We have updated the text in the Methods, Results, and Discussion session to reflect this.

- **METHODS:**
  - **(Pg 10):** *"We then performed repeated stratified 5-fold cross-validation as an additional check on the robustness of the model. This involved refitting the model on 80% of the cohort data and testing accuracy on the held out 20% for each of 10 5-fold random partitions, giving a total of 50 cross-validation folds/models, which are averaged to find the final model sequence."*
- **RESULTS:**
  - **(Pg 13):** *"Using repeated stratified 5-fold cross-validation (Supp. Fig. 2) as an alternative method to assess model robustness (both in terms of the sequence and uncertainty in the sequence), the maximum likelihood sequence is preserved with similar uncertainty in relative positions when visually compared to the bootstrapping method (Fig. 1B)"*
- **DISCUSSION:**
  - **(Pg 15):** "we explored the generalisability and robustness of the model using **two different validation methods**; bootstrap cross validation and **5-fold repeated stratified 5-fold cross-validation**. **These demonstrate** that even with a more conservative estimate of uncertainty, the sequence of atrophy is largely conserved (Fig. 1B **and Supp. Fig. 2**)."
  - **(Pg 15):** ***"****and after* ***cross validation*** *one can see (Fig. 1B* ***and Supp. Fig. 2)*** *that after the medulla there is uncertainty as to the exact ordering of the midbrain, thalamus, and ventral DC.****"***

4. Results: Is baseline EBM stage correlated with the PSP rating score at baseline? This is also very important.

We agree and have run this analysis including the figure in the supplementary material as Supp. Fig. 3. This shows that EBM stage is correlated with PSP rating score at baseline (β=1.14, 95% CI 0.8-1.44, p<0.001) and an adjusted R^2 of 0.18 (compared to the marginal R^2 for the fixed effects from the all-timepoints linear mixed model of 0.22). We have updated the text in the Methods, Results, and Discussion session to reflect this.

- **METHODS:**
  - **(Pg 11):** "To confirm that baseline EBM stage was also correlated with both PSPRS score and disease duration we fitted a linear model for each as a function of EBM stage."
- **RESULTS:**
  - **(Pg 14):** *"When fitting linear models for PSPRS score and disease duration versus predicted EBM stage on baseline scans only (Supp. Fig 3A / B respectively),* ***there was also a significant association albeit with a lower adjusted R2 (PSPRS vs EBM stage at baseline: β=1.14, 95% CI 0.84-1.44, p<0.001), adjusted R2 0.18****, disease duration vs EBM stage at baseline: (β=0.25, 95% CI 0.20-0.30, p<0.001, adjusted R2 0.39)."*

- **DISCUSSION:**
    - **(Pg16):** *"This is supported by the association between EBM stage and disease duration (both at all timepoints and only at baseline) in those subjects for which disease duration was recorded (Fig. 4B)"*

5. Results: It is known that neurodegenerative diseases have great heterogeneity. Is there are different subtypes in PSP-RS? The EBM was established on the probability of biomarkers. Thus, a great heterogeneity (different progress patterns) can cause fault progress sequences inference. We wonder that the SuStaIn subtype method (Young AL et al, 2018) could be useful in solving this problem. Maybe you can test whether there are different progress pattern using this method. Or explain this.

This is future work beyond the scope of this current study. We agree that PSP pathology is known to be associated with significant clinical heterogeneity and pathology distributions (and therefore likely heterogeneous sequences of atrophy progression) as operationalised in the 2017 Movement Disorder Society PSP clinical criteria and demonstrated in the Kovacs 2020 paper. However, PSP Richardson syndrome (PSP-RS) the most common, and "classical" clinical presentation is very strongly associated with underlying PSP pathology. For this study, as described in the Methods section (Pg 6: "_Data from individuals with a clinical diagnosis of possible or probable PSP-Richardson Syndrome_**"), we only selected cases with a possible or probable diagnosis of PSP-Richardson Syndrome to try and limit the spatiotemporal heterogeneity within the cohort. In further work we plan to investigate PSP phenotype heterogeneity using subtype and stage inference (SuStain) on a cohort that contains a wider range of clinical PSP syndromes (i.e. PSP-RS, PSP-P, PSP-PGF, PSP-SL, PSP-F, and PSP-CBS) to characterise the heterogeneity of atrophy and progression. This was not, however, the focus of this study.**

6. Results: We also want to know the diagnosis accuracy of PSP-RS using the ROI biomarkers directly. Is it higher or lower than EBM stage?

The difficulty in diagnosing PSP early is due to both the absence of biomarkers for underlying PSP pathology as well as there often being overlap initially in clinical symptoms and signs with conditions such as Parkinson's disease, Corticobasal Syndrome (CBS) and Multiple System Atrophy (MSA). The MDS 2017 criteria were specifically introduced to try and increase the sensitivity of clinical diagnosis early in the disease course.

The focus of this study using the EBM was not to diagnose PSP-RS early through differentiating it from other disorders, as all cases included already had a diagnosis clinically of PSP-RS and were compared with healthy controls. The focus was instead to try and identify brain regions that atrophy early in the disease course, and to build a staging model for those with a clinical diagnosis of PSP-RS. To try and differentiate PSP-RS from conditions that have a similar clinical presentation early in the disease course one would need to build a classifier (such as a SVM or individual EBMs for each disease) and train it on a range of atypical parkinsonian syndromes (PSP, CBD, MSA) as well as PD. We did, however, look at the accuracy of the model (90%) in differentiating cases from controls by

applying the threshold of reaching stage 2 in the sequence and present this on page 17 (although, but this was not the focus of the study).

7. Introduction: Third paragraph '…with additional involvement of the subcortical structures.' Should change 'subcortical structures' to 'cortical structures'.
Thank-you for spotting this. The manuscript has been updated.

Comments from the Scientific Editor:

-Please supply a graphical abstract. A graphical abstract is a visual representation of the central finding or methodology of the paper. It will be displayed in the table of contents. Authors should strive to make the graphical abstract informative, interesting, visually appealing, and straightforward (no caption needed, no abbreviations except gene names). Please upload as a separate high resolution image file (tiff preferred). The technical requirements for the graphical abstracts are:
 --size of the submitted image: 1200 pixels square at 300dpi
 --font of the text: Arial 12-16 point (smaller fonts will not be legible online)

A graphical abstract has now been provided.

-Please add an Abbreviated Summary (up to 50 words) of your paper that captures the main purpose and conclusions of the work. The summary should state the main results written in the third person ('[1st author surname] et al. report that …') and conclusion. This summary will be used in the contents list online and should not contain any abbreviations other than those on the accepted abbreviations list and accepted gene/protein names, making it accessible to the non-specialist. Please upload it as a Word document. Allowed abbreviations: AIDS; ANOVA; AMPA; ATP; A,T,C,G; CNS; CSF; CT; DNA; ECG; EEG; EMG; GABA; HIV; MRI; NMDA; PET; PCR; RNA.

Using event-based modelling, Scotton et al. report the probabilistic sequence of brain atrophy progression in Progressive Supranuclear Palsy. The resulting data-driven model allows individual patient staging at baseline MRI scan with potential utility for clinical trial enrichment.

-please report in the figure legend the test used and relevant test statistic values alongside p values (for example F or t values for ANOVAs and t-tests)
Figure legends have been updated.
