# Supplementary material for: A data-driven model of brain volume changes in progressive supranuclear palsy
Source: Brain Commun. 2022 Apr 14;4(3):fcac098. doi: 10.1093/braincomms/fcac098 (PMC9118104; doi:10.1093/braincomms/fcac098)
Supplement: fcac098_Supplementary_Data [file Revision 1 - Decision Letter.pdf › Revision 1 - Decision Letter.pdf]

Revision 1 – Decision Letter

25-Jan-2022

Dear Dr. Scotton,

It is a pleasure to accept your manuscript entitled "A data-driven model of brain volume changes in Progressive Supranuclear Palsy" for publication in Brain Communications. Any comments made by the reviewer(s) are included below.

The next step is for your paper to go through “First Look”. We will contact you again shortly if any final files or formatting changes are required for publication or if we have any queries.

If no changes are needed, your submission will be transferred to our Production Department and you will hear from the Production Editor in due course.

Video abstracts - Brain Communications encourages authors to provide a video abstract to accompany their article. These are hosted on the Brain/Brain Communications YouTube channel. If you are interested in producing a 5-min video abstract to promote your article, then please contact the Editorial Office (brcoms.editorialoffice@oup.com) for further details and guidelines.

Thank you very much for your contribution. On behalf of the Editors of Brain Communications, we look forward to your future contributions to the Journal.

Best wishes

Dr. Graciela Muniz Terrera
Associate Editor, Brain Communications

Professor Tara Spires-Jones
Founding Editor, Brain Communications

Reviewer(s)' Comments to Author:

Reviewer: 1 (Günter Höglinger)

Comments to the Author
all issues resolved

Reviewer: 2 (Minming Zhang)

Comments to the Author
Good job! This work really impressed us a lot. Hopefully there will be more studies of this kind in the future. This model does work.
